# Single-cell sequencing depicts tumor architecture and empowers clinical decision in metastatic conjunctival melanoma

**DOI:** 10.1038/s41421-024-00683-y

**Published:** 2024-06-11

**Authors:** Hanhan Shi, Hao Tian, Tianyu Zhu, Qili Liao, Chang Liu, Peng Yuan, Yongyun Li, Jie Yang, Chunyan Zong, Shichong Jia, Jing Ruan, Shengfang Ge, Renbing Jia, Peiwei Chai, Shiqiong Xu, Xianqun Fan

**Affiliations:** 1 Department of Ophthalmology, Shanghai Ninth People’s Hospital, Shanghai Jiao Tong University School of Medicine, Shanghai, China; 2 Shanghai Key Laboratory of Orbital Diseases and Ocular Oncology, Shanghai, China; 3 Center for Basic Medical Research and Innovation in Visual System Diseases of Ministry of Education, Shanghai Jiao Tong University School of Medicine, Shanghai, China; 4 State Key Laboratory of Molecular Biology, Shanghai Key Laboratory of Molecular Andrology, CAS Center for Excellence in Molecular Cell Science, Shanghai Institute of Biochemistry and Cell Biology, Chinese Academy of Sciences-University of Chinese Academy of Sciences, Shanghai, China; 5 Tianjin Eye Hospital, Tianjin Key Lab of Ophthalmology and Visual Science, Nankai University Affiliated Eye Hospital, Tianjin Eye Institute, Tianjin, China

**Keywords:** Eye cancer, Targeted therapies, Tumour angiogenesis

## Abstract

Conjunctival melanoma (CoM) is a potentially devastating tumor that can lead to distant metastasis. Despite various therapeutic strategies for distant metastatic CoM, the clinical outcomes remain unfavorable. Herein, we performed single-cell RNA sequencing (scRNA-seq) of 47,017 cells obtained from normal conjunctival samples (*n* = 3) and conjunctival melanomas (*n* = 7). Notably, we noticed a higher abundance of cancer-associated fibroblasts (CAFs) in tumor microenvironment (TME), correlated with enhanced angiogenic capacity and increased VEGFR expression in distal metastatic CoM. Additionally, we observed a significant decrease in the proportion of total CD8^+^ T cells and an increase in the proportion of naive CD8^+^ T cells, contributing to a relatively quiescent immunological environment in distal metastatic CoM. These findings were confirmed through the analyses of 70,303 single-cell transcriptomes of 7 individual CoM samples, as well as spatially resolved proteomes of an additional 10 samples of CoMs. Due to the increase of VEGFR-mediated angiogenesis and a less active T cell environment in distal metastatic CoMs, a clinical trial (ChiCTR2100045061) has been initiated to evaluate the efficacy of VEGFR blockade in combination with anti-PD1 therapy for patients with distant metastatic CoM, showing promising tumor-inhibitory effects. In conclusion, our study uncovered the landscape and heterogeneity of the TME during CoM tumorigenesis and progression, empowering clinical decisions in the management of distal metastatic CoM. To our knowledge, this is the initial exploration to translate scRNA-seq analysis to a clinical trial dealing with cancer, providing a novel concept by accommodating scRNA-seq data in cancer therapy.

## Introduction

Conjunctival melanoma (CoM) is a sight- and life-threatening malignancy arising from the bulbar and palpebral conjunctiva and from the caruncle. CoM is obscure due to its undistinguishable origin, lack of knowledge of the tumor microenvironment (TME), and high metastatic death rate even after standard therapy^1^. Distant metastasis threatens prognosis of CoM patients, and the survival time of patients after systemic metastasis is much shorter than that of localized CoM patients^2^. Compared to CoM patients in Western countries, patients in China present with more aggressive clinical behaviors and worse prognoses^3^. Higher 5 and 10 year tumor-related mortality were observed in China (30.5% and 37.4%, respectively) than those in the Netherlands (13.7% and 28%, respectively)^3,4^. Hence, it is necessary to reveal tissue architecture, distinguish tumor origin and analyze heterogeneity of the TME to manage therapeutic strategies, especially for Chinese CoM patients.

Under most circumstances, CoMs arise from primary acquired melanosis (PAM) with atypia, accounting for an estimated 53%–75% of cases^5–7^. The other CoM develops from a conjunctival nevus or can be formed de novo without any preceding lesion. Notably, increasing evidence has demonstrated a common genetic kinship between conjunctival and cutaneous melanoma (e.g., *BRAF, NRAS, NF1, KIT*)^8^ while also differentiating it from uveal melanoma (e.g., *GNAQ/11, BAP1, SF3B1*)^9^. CoMs originate from melanocytes in the basal layer of the epithelium of the conjunctival membrane. They show invasion of atypical melanocytes from the overlying conjunctival epithelium penetrating the basement membrane into the subepithelial connective tissue (substantia propria). Both internal factors (genetics, pre-exiting lesions, and melanin pigments) and external factors (UV radiation) contribute to the development of CoM. However, a systematic profiling of CoM at single-cell resolution has not been established. Further molecular characteristics are needed to enable an in-depth understanding of CoM biological behaviors.

Herein, we initially characterized the comprehensive cellular landscape using single-cell RNA sequencing (scRNA-seq) at different tumor stages of CoM. Significantly, an elevated prevalence of cancer-associated fibroblasts (CAFs) within the TME was observed, which was found to be associated with heightened angiogenic potential and increased vascular endothelial growth factor receptor (VEGFR) expression in distal metastatic CoM. Furthermore, a notable reduction in the ratio of CD8^+^ T cells and an increase in the proportion of CD8^+^ T naive cells were observed, thereby contributing to a relatively inactive immunological milieu in distal metastatic CoM. These results were validated by analyzing 70,303 single-cell RNA transcriptomes from 7 independent samples, along with spatially resolved proteome analyses in an additional 10 samples of CoMs. Based on these findings, we launched a clinical trial using VEGFR blockade combined with anti-PD1 therapy for distal metastatic CoM patients, which triggered efficient therapeutic efficacy. In summary, our study reports the tissue architecture and unique TME of CoM for the first time and provides a novel concept by integrating scRNA-seq data in the decision of cancer therapy.

## Results

### Single-cell profiling of benign conjunctiva and the CoM microenvironment

**Fig. 1:**
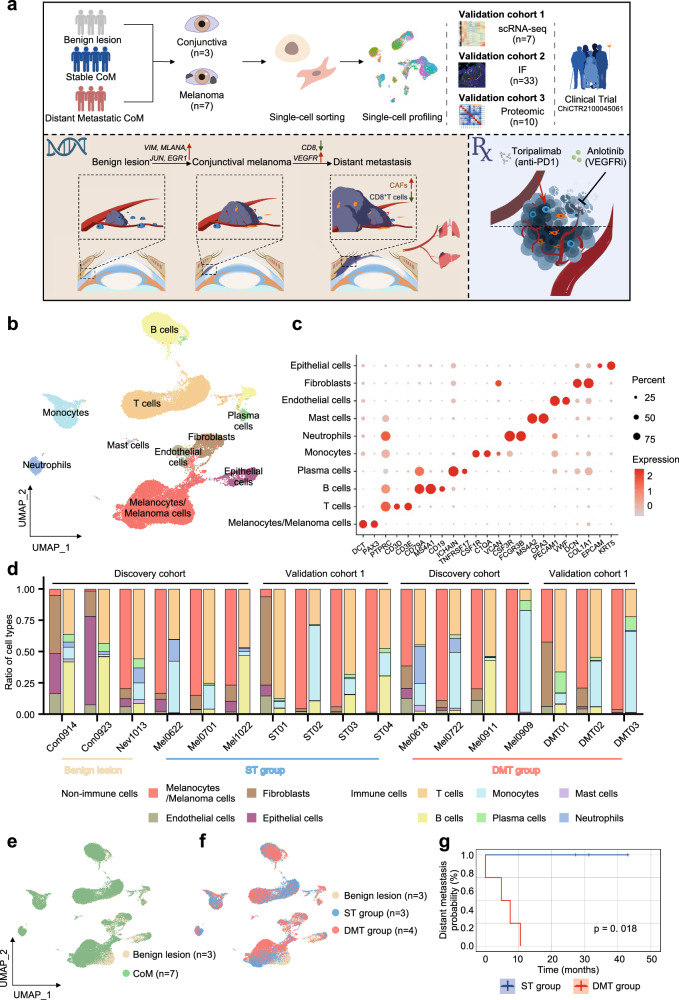
Diversity of cell types in CoM and nonmalignant conjunctival samples annotated by scRNA-seq. **a** Schematic representation of the experimental strategy, indicating the collection and administration of samples from patients with CoM and nonmalignant conjunctiva. **b** UMAP plot illustrating the annotation of diverse cell types in CoM and nonmalignant conjunctival tissues. **c** Dot plot showing expression levels of specific cell marker genes in different cell types. The size of the dot suggests the proportion of cells expressing the marker genes. The spectrum of colors indicates the mean expression levels of the marker genes. **d** Bar plots indicating the proportion of annotated cell types in 3 nonmalignant samples (2 conjunctival samples and 1 conjunctival nevus), 14 primary CoM samples (discovery cohort: *n* = 7, validation cohort 1: *n* = 7). The annotated cell types and colors are presented in the bottom. **e**, **f** UMAP plot showing cell origins by color: benign or melanoma origin (**e**) benign lesion in white and melanoma in green, and ST or DMT origin (**f**) ST group in blue and DMT group in red. **g** Progression-free survival based on distant metastasis in CoM patients from ST (blue) and DMT (red) group enrolled in the scRNA-seq cohort.

To systematically interrogate the origin and tumor landscape of CoM, we collected conjunctiva samples (*n* = 3) and conjunctival melanoma samples (*n* = 7), which were subsequently subjected to scRNA-seq analysis. Primary tumor tissues from CoM patients who did not exhibit known metastasis within a follow-up period of at least 12 months were categorized as the stable tumor group (ST group, *n* = 3), while primary tumor tissues from patients who developed distant metastasis were categorized as the distant metastatic tumor group (DMT group, *n* = 4). Detailed clinical and pathological information, including the origin, clinical and pathological characteristics, driver mutation and prognosis, is provided in Supplementary Table S1. We expanded our study by including an additional cohort of conjunctival melanoma samples (*n* = 7) for scRNA-seq validation (validation cohort 1). Furthermore, a larger number of patients were included as validation cohort 2 for analyses of expression level of the targeted protein by immunofluorescence (IF)_staining. This IF cohort consisted of 13 benign conjunctival lesions, 18 primary CoM lesions (including 10 stable CoM and 8 distal metastatic CoM), and 2 distal metastatic lesions from patients with distant metastatic CoM (1 subcutaneous metastatic sample and 1 lung metastatic sample). Additionally, we included 10 additional CoM samples for spatial proteome profiling (validation cohort 3) to validate the molecular aberrations identified (Fig. 1a). All the clinical information of CoM patients in validation cohort was concluded in Supplementary Table S2.

A total of 47,017 single cells were acquired from 10 samples and subjected to scRNA-seq (Supplementary Fig. S1a). Of these cells, 14,939 cells (31.8%) were from benign conjunctival tissues, and the other 32,078 cells (68.2%) were from tumor-related tissues (Supplementary Fig. S1b, c and Table S3). Dimensional reduction analysis was used to show the heterogeneity of melanocytes/melanoma cells, stromal cells and other nonmalignant cell types, in which cells were clustered and defined according to the established gene markers (Fig. 1b). Specifically, the annotated cell types in the TME and nonmalignant tissues included melanocytes/melanoma cells (marked by *DCT* and *PAX3*), T cells (marked by *PTPRC*, *CD3D* and *CD3E*), B cells (*CD79A*, *MS4A1* and *CD19*), plasma cells (*ICHAIN* and *TNFRSF17*), monocytes (*CSF1R*, *C1QA* and *VCAN*), neutrophils (*CSF3R* and *FCGR3B*), mast cells (*MS4A2* and *CPA3*), endothelial cells (*PECAM1* and *VWF*), fibroblasts (*DCN* and *COL1A1*) and epithelial cells (*EPCAM* and *KRT5*) (Fig. 1c), following the standard from reported scRNA-seq studies^10,11^.

Notably, we noticed a similar expression pattern between melanoma cells and conjunctival melanocytes following dimensional reduction. Herein, we have applied two complementary methods to distinguish melanoma cells and nonmalignant melanocytes. First, we inferred chromosomal copy number variations (CNVs) in each single cell according to the averaged expression profiles across chromosomal intervals^12^. These inferred CNVs separated malignant cells from benign melanocytes based on normal karyotypes (Supplementary Fig. S2a). In addition, we recognized malignant CoM cells according to the expression of a panel of melanoma-associated marker genes (*S100A1*, *PRAME*, *TYRP1*, *SOX10*, *MLANA* and *VIM*). Conclusively, the characterization of malignant melanoma cells vs nonmalignant melanocytes was able to be confirmed by both CNV and melanoma marker gene expression analyses (Supplementary Fig. S2b). Moreover, we compared differentially expressed genes between well-defined melanocytes and melanoma cells, further underscoring a distinct transcriptional pattern between melanoma cells and benign melanocytes during CoM progression (Supplementary Fig. S2c).

With the definition of individual cell subset types as well as the clinical subset types according to the probability of developing distant metastasis, the proportion of cell types in each sample was analyzed. The cellular proportion in benign lesions, ST CoM lesions and DMT CoM lesions was quite different, while the cell subset types were shared, comprising melanocytes/melanoma cells, epithelial cells, endothelial cells, fibroblasts, T cells, B cells, plasma cells, neutrophils, mast cells and monocytes (Fig. 1d). Nevus presented the similar cell type composition with CoM samples because of its origin. Uniform manifold approximation and projection (UMAP) analysis visualized the distribution of benign lesions and CoM lesions, which were further divided into the ST group and DMT group according to the follow-up of patients from whom these samples were collected from (Fig. 1e–g; Supplementary Fig. S1b, c). In the validation cohort 1, a total of 70,303 single cells were obtained from 7 distinct samples and underwent scRNA-seq, revealing the presence of tumor heterogeneity (Supplementary Fig. S3). Collectively, we observed remarkable heterogeneity among individual tumors in their composition, both in the ST and DMT groups.

### Transcriptional trajectory analysis identified the activation of several oncogenic signaling pathways during tumorigenesis and progression of CoM

**Fig. 2:**
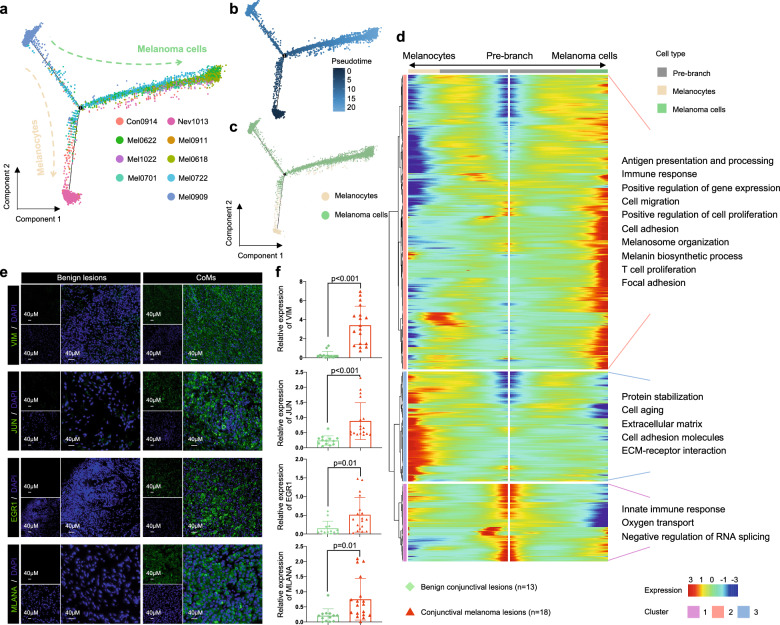
Differential gene expression signatures reveal triggers for tumorigenesis and metastasis of CoM. **a** Transcriptional trajectory analysis of 3041 melanocytes and 11,964 melanoma cells from all conjunctival samples colored by patient ID. **b** The transcriptional trajectory of a differential process is visually represented using color-coded states, wherein each data point corresponds to an individual cell. The range of colors employed signifies the diverse states of both melanocytes and melanoma cells. Both branches emanate from a shared progenitor state (located on the left), with the right branch denoting the progression towards melanoma cells and the downward branch indicating the transition towards melanocytes. **c** Pseudotime analysis was employed to compare the distribution between melanocytes and melanoma cells. Each data point refers to an individual cell. Melanocytes are labeled in white while melanoma cells in green. **d** Heatmap shows upregulated or downregulated genes in the differentiation process. The differentially expressed genes (rows) along the pseudotime (columns) were hierarchically clustered into three subclusters. The representative enriched pathways of each subcluster are provided. **e** Representative images of IF staining in FFPE tissues from validation cohort, indicating VIM-, JUN-, MLANA- and EGR1-positive cells, in benign lesion and CoM tissue sections. Scale bars, 40 μm. **f** Averaged expression of VIM, JUN, MLANA and EGR1 in nonmalignant conjunctival lesions (green) and CoM tissues (red) from validation cohort, based on IF staining results. All statistical analyses are two-tailed unpaired Student’s *t*-test, *n* represents the number of samples from patients.

It is worth mentioning that transcriptional states of both skin cutaneous melanoma (SKCM) and uveal melanomas have been previously elucidated through single-cell sequencing^13,14^. However, the heterogeneity of landscape and transcriptional trajectory of CoM remains uncertain. To fully address the origin and elucidate the transcriptional states across the oncogenesis and progression of CoM, we applied transcriptional trajectory analysis on 3041 nonmalignant melanocytes and 11,964 melanoma cells. The inferred trajectory of state transitions exhibits two distinct lineages, displaying a bifurcated configuration from the initial state to the final states. These lineages can be further categorized into three distinct clusters based on their transcriptional states (Fig. 2a). Both lineages originate from a common progenitor state and subsequently diverge following intermediate states, with one lineage progressing towards melanoma cells and the other differentiating into melanocytes (Fig. 2b, c). Melanocytes were predominantly presented in one terminal state (cluster 2), while the melanoma cells were distributed over the undifferentiated progenitor state (cluster 1) and the other identified terminal differentiated state (cluster 3) (Supplementary Fig. S4a and Table S4). We then analyzed the trajectories of each cluster individually with Monocle 2 using branched expression analysis modeling (BEAM) and hierarchical clustering to identify genes enriched across states. Along the trajectory, the gene signature of cluster 3 in melanoma cells was characterized as the activation of certain functional pathways, including the cell adhesion pathway (*VIM*), immune response signaling pathway (*JUN*, *EGR1*), and melanosome organization signaling pathway (*MLANA*) (Fig. 2d, e; Supplementary Table S5). Concordantly, IF analysis of validation cohort 2, comprising 13 benign conjunctival lesions and 18 primary CoM lesions, revealed heightened expression levels of these marker genes in malignant conjunctival melanoma samples (Fig. 2e, f).

In order to delve deeper into the progression of CoM, Monocle algorithm was utilized to conduct pseudotime analysis on melanoma cells, with the aim of profiling their evolutionary trajectories in both ST and DMT groups (Supplementary Fig. S4b). The pseudotime orders of transcriptional states were employed to classify a total of 3 clusters of cells into 3 main branches (Supplementary Fig. S4c, d). At the subset level categorized by clinical outcome, it was observed that melanoma cells from the ST group were primarily concentrated in clusters 1 and 3, whereas cluster 2 was predominantly contributed by the DMT group (Supplementary Fig. S4e, f and Table S6). In order to gain a deeper understanding of the factors driving progression in CoM, marker genes along the progression trajectories in BEAM were utilized and compared (Supplementary Fig. S4g). Notably, we observed a series of activated signaling pathways in cluster 2, including glycolysis (*P* < 0.001), and glutathione metabolism (*P* = 0.03). Notably, these signaling pathways also support distal metastasis of SKCM and other mucosal melanomas^15–17^. Collectively, these observations elucidate the landscape during the commencement and progression of the CoM, potentially indicating a comparable pattern of transformation between CoM and other variants of melanoma.

### CAFs support distant CoM metastasis by enhancing angiogenesis

**Fig. 3:**
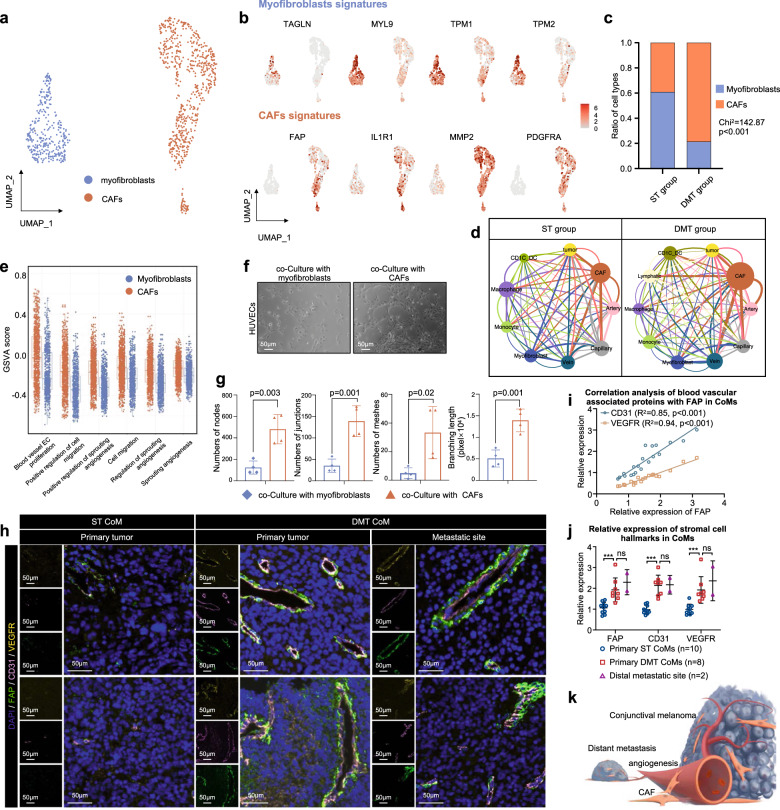
Transcriptomic features of CAFs indicate their potential role in promoting distant metastasis by enhancing angiogenesis. **a** UMAP plot showing the subtypes of CAFs derived from ST and DMT patients, colored by cell type, myofibroblasts in blue and CAFs in orange. **b** UMAP plot illustrating expression of marker genes of myofibroblasts (*TAGLN*, *MYL9*, *TPM1* and *TPM2*), and CAFs (*FAP*, *IL1R1*, *MMP2* and *PDGFRA*). The spectrum of colors indicates the mean expression levels of the marker genes. **c** Bar plots showing the proportion of CAFs and myofibroblasts in ST and DMT samples. The difference was statistically significant. All statistical analyses are Chi-square tests. **d** Communication networks of CAFs, vascular endothelial cells, tumors and the immune microenvironment in the ST and DMT groups. **e** Box and whisker plot of angiogenesis GSVA score in myofibroblasts and CAFs. **f** HUVEC tube formation assay showing the effect of myofibroblasts and CAFs on HUVEC tubule formation. Scale bars, 50 μm. **g** Statistics of the numbers of nodes, junctions, meshes and the branching length in tube formation experiments. *n* = 4. Data are presented as means ± SD. Two-tailed unpaired Student’s *t*-test. The data of the HUVECs cocultured with myofibroblasts group was set to 1. **h** IF staining of FAP (CAF marker), VEGFR and CD31 in the ST group and DMT group. Distal metastatic sites from DMT CoM patients were included in DMT group. The representative images were displayed (ST group: primary CoM tissues, *n* = 2, left; DMT group: primary CoM tissues, *n* = 2, middle; distal metastatic lesions, *n* = 2, right). Scale bars, 50 μm. **i** Correlation analysis of blood vascular-associated proteins with FAP in CoMs (CD31 in blue, VEGFR in orange). **j** Relative expression of stromal cell hallmarks in primary ST CoMs, primary DMT CoMs, and distal metastatic sites according to the results of IF staining (One-way ANOVA). **k** Schematic of CAFs showing their possible role in promoting distant metastasis by enhancing angiogenesis.

Stromal fibroblasts are an important component of the TME, contributing to tumor progression as well as immunotherapy resistance^18^. We then detected and explored the features of stromal fibroblasts, including CAFs and myofibroblasts, according to the expression of marker genes of CAFs (*FAP*, *IL1R1*, *MMP2* and *PDGFRA*) and myofibroblasts (*TAGLN*, *MYL9*, *TPM1* and *TPM2*), as previously described (Fig. 3a, b; Supplementary Fig. S5 and Table S7)^10,19^. More CAFs were observed in the DMT group than in the ST group (discovery cohort: *χ*^2^ = 142.87, *P* < 0.001, shown in Fig. 3c; validation cohort 1: *χ*^2^ = 11.46, *P* < 0.001, shown in Supplementary Fig. S5f–h). The large proportion of CAFs in the DMT group suggested an oncogenic role of CAFs in DMT samples of CoM.

To explore the mechanism by which CAFs contribute to distant metastasis, we analyzed and compared the expression of CAFs and myofibroblasts. The genes that exhibited differential expression were found to be significantly enriched in angiogenesis pathway (*P* < 0.001, Supplementary Fig. S5a, b). Consistently, expression levels of angiogenesis-associated genes were markedly elevated in CAFs, including *IGF1* (*P* < 0.001), *IGF2* (*P* < 0.001), *VEGFA* (*P* < 0.001), and *VEGFB* (*P* < 0.001) (Supplementary Fig. S5c, i). The cell types in vascular endothelial system, including artery, vein and capillary, were defined by marker genes to fully address the correlation between CAFs and endothelial cells (Supplementary Fig. S5d)^10,19^. As expected, CAFs showed a strong positive correlation with vascular endothelial systems, including increased CAF–artery, CAF–vein, and CAF–capillary interactions (Fig. 3d; Supplementary Fig. S5e). Moreover, elevated GSVA scores of a series of angiogenic signaling pathways were observed in CAFs, including endothelial cell proliferation (*P* < 0.001), migration (*P* < 0.001), and sprouting (*P* < 0.001) (Fig. 3e). These results suggested that CAFs share many angiogenic markers with blood vessels, which was consistent with previous studies^20^.

In order to isolate primary CAFs and myofibroblasts from clinical distal metastatic samples of CoM, the tumor tissues underwent differential trypsinization following established protocols^21^. Subsequently, fibroblast subpopulations (CAFs and myofibroblasts) were characterized by fluorescence-activated cell sorting (FACS). CAFs were identified as fibroblasts expressing fibroblast activation protein (FAP), whereas myofibroblasts were identified as fibroblasts with low expression of FAP (Supplementary Fig. S5j). The distinguished cells were further verified by IF staining against markers of CAFs (FAP) and myofibroblasts (MYL9) (Supplementary Fig. S5k). As evaluated by Matrigel tube formation assays, CAFs enhanced the tube-formation ability of human umbilical vein endothelial cells (HUVECs) (Fig. 3f, g). We further speculated that CAFs promote angiogenesis and form fibrovascular niches, both of which play a positive role in tumor progression. To fully recapitulate the molecular characteristics of primary and distant metastatic CoM, we collected primary and distal metastatic lesions from ST and DMT patients in the validation cohort 2, and evaluated the expression of FAP, CD31 and VEGFR. In alignment with previous observations, CAFs often wrapped around endothelial cells to form fibrovascular niches (Fig. 3h)^22^. Specifically, positively correlated expression between FAP and blood vessel markers (*CD31* and *VEGFR*) was observed (Fig. 3i, j). Collectively, these results suggested that CAF activation during angiogenesis contributes to CoM distant metastasis (Fig. 3k).

### A relatively quiescent immune ecosystem is associated with distant metastatic CoM

**Fig. 4:**
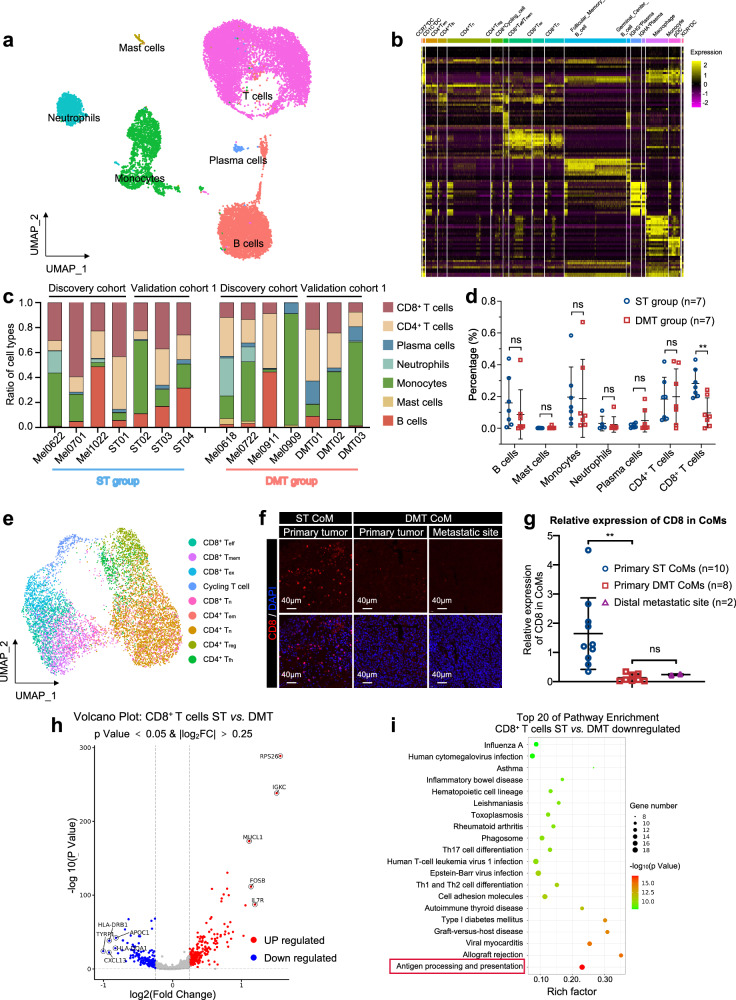
scRNA-seq profiling of the tumor immune microenvironment in CoM. **a** UMAP plot showing immune cell types in CoM samples, labeled in different colors. **b** Heatmap plot showing the expression levels of marker genes in each subtype of immune cells. **c** Bar plots showing the proportion of annotated cell types in 7 samples from the ST group and 7 samples from the DMT group. **d** Proportion of each immune cell subset in the ST group and DMT group from both discovery cohort and validation cohort 1. Two-tailed unpaired Student’s *t*-test. **e** UMAP plot showing the T cell subsets from all 7 CoM samples in discovery cohort, labeled in different colors. **f** IF staining with CD8 antibody in CoM tissue sections and distal metastatic sites, from ST and DMT groups separately. Distal metastatic sites from DMT CoM patients were included in DMT group. The representative images were displayed (ST group: primary CoM tissues, *n* = 2, left; DMT group: primary CoM tissues, *n* = 2, middle; distal metastatic lesions, *n* = 2, right). Scale bars, 40 μm. **g** Average expression of CD8 in the primary ST CoMs, primary DMT CoMs, and distal metastatic sites according to the results of IF staining (One-way ANOVA). **h** Volcano plots of differentially expressed genes in CD8^+^ T cells between ST group and DMT group. **i** Bubble plots of downregulated genes in CD8^+^ T cells between ST group and DMT group enriched pathways. The top 20 processes were displayed.

Signals from the immune microenvironment educate adaptive plasticity of tumor cells, playing a vital role in the determination of clinical outcome^23^. To profile the tumor immune microenvironment, we generated a UMAP plot of immune cells and examined the average gene expression across tumor samples to identify hierarchical clusters (Fig. 4a)^24^. The identified immune cells included T cells (*PTPRC*, *CD3E* and *CD3D*), B cells (*CD79A*, *MS4A1* and *CD19*), mast cells (*MS4A2* and *CPA3*), monocytes (*CSF1R*, *C1QA* and *VCAN*), neutrophils (*CSF3R* and *FCGR3B*), and plasma cells (*ICHAIN* and *TNFRSF17*) (Fig. 4b). Notably, the proportions varied with substantial heterogeneity between the ST and DMT groups in both discovery cohort and validation cohort 1 (Fig. 4c). We observed very few infiltrations of mast cells and neutrophils in all CoMs. In contrast, other immune cell compositions varied between the DMT group and the ST group. The infiltration levels of B cells and T cells in DMT samples (*n* = 7) were lower than those in ST (*n* = 7) samples, especially CD8^+^ T cells, in both discovery cohort and validation cohort 1 (total: *P* = 0.003, discovery cohort: *P* = 0.002, validation cohort 1: *P* = 0.05, shown in Fig. 4d; Supplementary Fig. S6a, b). In summary, these results suggested that the varied immunological microenvironment is associated with metastatic outcomes in CoM patients, despite such remarkable heterogeneity.

We further applied dimensional reduction analysis to illustrate the heterogeneity of T cells. As shown in the UMAP plot, T cells were clustered into 9 subtypes by average expression levels of marker genes (Fig. 4e). Consistent with the scRNA-seq results, a low proportion of CD8^+^ T cells was observed in distant metastatic CoM tissues, including primary and metastatic lesions in the validation cohort 2 (Student’s *t*-test, *P* = 0.003; Fig. 4f, g). CD8^+^ T cells in the ST and DMT samples exhibited distinct transcriptomic profiles, as evidenced by upregulation of 216 genes and downregulation of 173 genes in CD8^+^ T cells of DMT samples as compared to ST samples (Fig. 4h). Concordantly, these downregulated genes were enriched in immune-related pathways, including antigen processing and presentation, T cell differentiation and autoimmune diseases (Fig. 4i). Collectively, these data suggested that the immunological ecosystem in distal metastatic CoM exhibited decreased infiltration levels of CD8^+^ T cells, contributing to a relatively inactive immunological milieu in distal metastatic CoMs.

### Functional CD8^+^ T cells were excluded in distant metastatic CoM

**Fig. 5:**
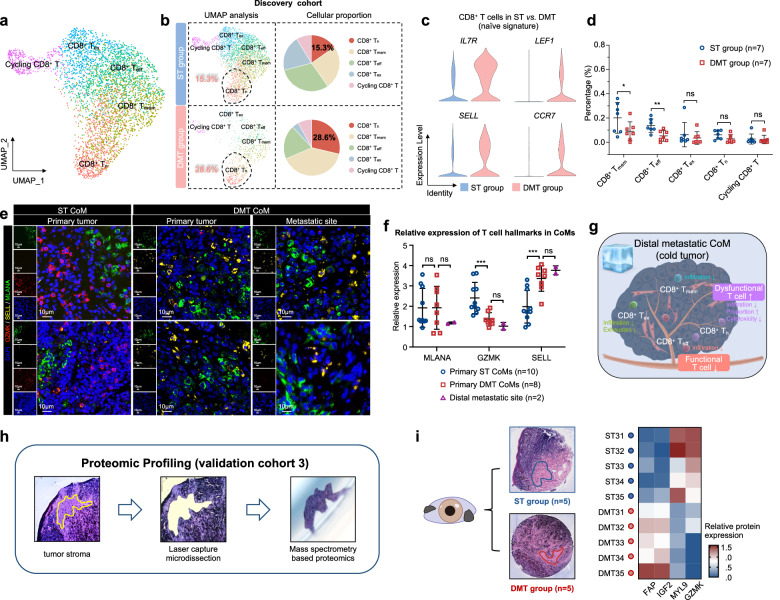
CD8^+^ T cells play crucial roles in the distinctive immune microenvironment of distant metastatic CoM. **a** UMAP plot showing the CD8^+^ T cell subsets from all 7 CoM samples, labeled in different colors. **b** The proportions of specific subsets of CD8^+^ T cells between the ST group and DMT group were analyzed (left). Pie charts showing the proportion of CD8^+^ T_n_ cells in ST and DMT groups (right). **c** Violin plot indicating the expression of naive signature of CD8^+^ T cells in ST and DMT groups. **d** The infiltration percentage of specific subsets of CD8^+^ T cells between the ST group (*n* = 7) and DMT group (*n* = 7) in both discovery cohort and validation cohort 1 was analyzed. Two-tailed unpaired Student’s *t*-test. **e** IF staining validating the characteristics of immune microenvironment in ST and DMT samples. Distal metastatic sites from DMT CoM patients were included in DMT group. The representative images were displayed (ST group: primary CoM tissues, *n* = 2, left; DMT group: primary CoM tissues, *n* = 2, middle; distal metastatic lesions, *n* = 2, right). Scale bars, 10 μm. **f** Relative expression of MLANA, GZMK, and SELL in primary ST CoMs, primary DMT CoMs, and distal metastatic sites according to the results of IF staining (One-way ANOVA). **g** Schematic diagram showing changes of CD8^+^ T cells in distant metastatic CoM. **h** FFPE tissue was sectioned and stained with hematoxylin and eosin (H&E) to identify histomorphological features. Tissues were then excised using LCM and prepared for LC-MS/MS-based proteomics. **i** Differentially expressed proteins between ST group (*n* = 5) and DMT group (*n* = 5), patient of origin is annotated on the left.

We then applied dimensional reduction analysis to further illustrate heterogeneity of CD8^+^ T cells. As shown in the UMAP plot, CD8^+^ T cells were clustered into 5 subtypes by average expression of marker genes, according to previous approaches (Fig. 5a)^24^.

To demonstrate the detailed functional subset of CD8^+^ T cells, we compared the cell proportions between the ST and DMT groups. Specifically, a significant elevation in the cellular proportion of CD8^+^ naive T cells (T_n_) was observed in the DMT group (discovery cohort: 15.3% in ST vs 28.6% in DMT, *χ*^2^ = 73.13, *P* < 0.001; validation cohort 1: 16.4% in ST vs 20.5% in DMT, *χ*^2^ = 17.92, *P* < 0.001; shown in Fig. 5b; Supplementary Fig. S6c). CD8^+^ T cells in the DMT group showed increased expression of *IL7R* (*P* < 0.001), *LEF1* (*P* < 0.001), *SELL* (*P* < 0.001) and *CCR7* (*P* < 0.001), suggesting a naive and dysfunctional phenotype (Fig. 5c). Since the diminished cytotoxicity of naive T cell population, which has been observed in metastatic tumors exhibiting reduced infiltration of conventional functional CD8^+^ T cells^25^, indicating that T cell reprogramming plays a crucial role in distal metastatic CoMs.

To further assess the functional effects of the immunological ecosystem in DMT samples, we isolated CD8^+^ T cells and compared the infiltration of each subset in separated samples from the ST and DMT groups. Most of the CD8^+^ T cell subsets showed lower infiltration levels in the DMT group than in the ST group, and the fractions of CD8^+^ effector T (T_eff_) cells (*P* = 0.01) and CD8^+^ memory T (T_mem_) cells (*P* = 0.03) were significantly lower in the DMT group (Fig. 5d; Supplementary Fig. S6d, e). Notably, the CD8^+^ T_eff_ cells in DMT group presented with increased expression of *PDCD1* (therapy target of anti-PD1) (Supplementary Fig. S6f), which further underscores the dysfunction of T cell population in the metastatic microenvironment of CoMs. However, no obvious difference was observed in CD4^+^ T cells (Supplementary Fig. S6g).

Additionally, the distinctive features of CD8^+^ T cells, including the elevated proportion of dysfunctional subset (measured by naive T cell marker *SELL*^24^) and decreased infiltration of functional subsets (measured by effective T cell marker *GZMK*^24^), were validated by IF staining in the ST and DMT (primary and metastatic lesions) groups (Fig. 5e, f). Collectively, the elevated proportion of dysfunctional T_n_ cells and low infiltration of CD8^+^ T_eff_/T_mem_ cells contribute to the quiescent microenvironment, referring to the distal metastatic CoM as a “cold” tumor with functional T cells excluded (Fig. 5g).

### Proteomic profiles of histomorphological niches highlight regionally unique biological processes in TME and validate scRNA-seq findings

In order to investigate proteomic patterns of the TME, we have performed spatial proteomic analysis in a cohort of 10 well sampled formalin-fixed paraffin embedded (FFPE) primary CoMs. For each single case, the stromal region of CoM was defined by consensus annotations by two board certified pathologists from the Department of Pathology in Shanghai Ninth People’s Hospital and laser capture microdissection (LCM) was used to excise regions of TME, as previously described^26^. In total, we have isolated 10 CoM samples (*n* = 5 for ST, *n* = 5 for DMT) for analysis by LC-MS/MS resulting in quantification of a total of 3514 proteins, with 66 upregulated and 48 downregulated proteins (Fig. 5h; Supplementary Fig. S7a).

Importantly, DMT samples demonstrated increased angiogenic capabilities with increased expression of CAF markers (*FAP*^27^ and *IGF2*^28^), decreased expression of myofibroblast marker (*MYL9*^19^), and reduced expression of functional T cell marker (CD8^+^ T_eff_ cells marked by *GZMK*^24^), aligning with the findings of the discovery cohort. These findings align with the observed increase in CAF-mediated angiogenesis and dysfunctional T cells within the TME of metastatic CoMs (Fig. 5i; Supplementary Table S8).

### VEGFR blockade combined with anti-PD1 therapy triggers clinical efficacy in distant metastatic CoM patients

Prior research has indicated that metastatic CoM and metastatic mucosal melanoma exhibited limited response to anti-PD1 monotherapy^29,30^. Conversely, the combination of anti-angiogenic agents with anti-PD1 has shown a promising safety profile and has demonstrated significant antitumor activity in various solid tumors, especially in cutaneous melanoma^31^. Following the above data, we launched a clinical trial for distant metastatic CoM patients (ChiCTR2100045061, http://www.chictr.org.cn/index.aspx). Clinical details for the patients enrolled were collected and are presented in Supplementary Table S9.

**Fig. 6:**
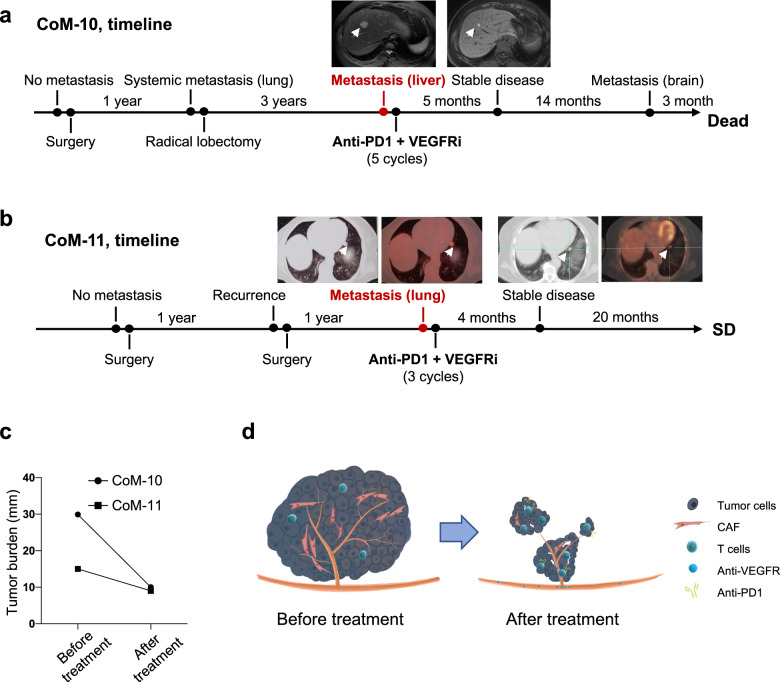
Clinical trial of VEGFR blockade combined with anti-PD1 therapy in systemically metastasized CoM patients. **a** Chronologic changes in the radiology of patient CoM-10 treated with VEGFR blockade combined with anti-PD1 therapy. **b** Diagram of CoM progression and treatment for patient CoM-11. The tumor was confirmed to recur and progress rapidly. With VEGFR blockade combined with anti-PD1 therapy, no progression was observed. **c** Chronologic changes in the tumor burden of patients. **d** Schematic drawing of the likely underlying mechanisms of the clinical trial.

In order to investigate the clinical effectiveness of combining VEGFR blockade (Apatinib) with anti-PD1 (Camrelizumab) therapy, a cohort of patients with distal metastasis was recruited and documented. Patient CoM-10 received VEGFR inhibitor (VEGFRi) and anti-PD1 treatment following the identification of multiple liver metastases through PET/CT. Following a standard treatment regimen for a duration of 5 months, imaging evaluation of this patient revealed a state of stable disease (Fig. 6a). The patient progressed after 14 months of stable disease and experienced a 22.42 month follow-up from stable disease till death as a result of brain metastasis. Patient CoM-11 was diagnosed with distant metastasis by PET/CT showing metastasis to the upper lobe and lower lobule of the left lung in 2021. After 3 courses of anti-VEGFR and anti-PD1 therapy, there was no evidence of disease progression (Fig. 6b). Up to the latest follow-up in Aug 2023, the patient was alive with neoplasm. Tumor burden was calculated and compared. According to the RECIST 1.1 criteria, decreased tumor burden was observed in both patients after VEGFR blockade combined with anti-PD1 therapy (Fig. 6c).

To further assess the clinical efficacy of immune therapy and targeted therapy, we analyzed the clinical features and treatment information of distant metastatic CoM patients between Jan 2020 and Aug 2023 in our center. Detailed clinical and pathological information is presented in Supplementary Table S10, as well as the treatment procedure. Of all 11 distant metastatic CoM patients, 5 patients did not receive systemic treatment following local excision and had a median survival time of 3.91 months (in blue). Four patients received immune therapy with anti-PD1, with a median survival time of 5.67 months (in red). The aforementioned 2 patients who enrolled in the clinical trial and received anti-VEGFR and anti-PD1 therapy exhibited prolonged prognoses (median survival time = 23.71 months, in green, *P* = 0.02 according to the log-rank *P* test, Supplementary Fig. S8a). Collectively, VEGFR blockade combined with anti-PD1 therapy could be considered a novel therapeutic strategy for CoM patients with distant metastasis (Fig. 6d).

## Discussion

Despite advancements in diagnostic and treatment modalities, clinical outcome of CoM continues to be unsatisfactory due to frequent occurrence of distant metastasis, even following orbital exenteration surgery^32^. Considering the distinct genetic backgrounds and mutational profiles observed in cutaneous, uveal, and conjunctival melanoma, it is imperative to modify therapeutic approaches based on the specific genetic characteristics of each subtype. Moreover, the lack of a comprehensive comprehension regarding the fundamental mechanisms dictating the advancement of CoM impedes the translational implementation of potential targeted therapeutic interventions for managing CoM. Herein, our scRNA-seq study revealed a high-resolution depiction of the complex ecosystem of tumor, stromal fibroblasts, and immune cells and empowers clinical decision-making in DMT CoM.

The transcriptomic signatures of CoM were illustrated. One of our observations was the identification of key genes contributing to the evolution of transcriptional states, including *VIM*, *JUN*, *MLANA* and *EGR1*. These elevated oncogenic signaling pathways were also observed in malignant transformation of SKCM^23^ and uveal melanoma^14,33^, suggesting a shared genetic kinship of the initiation among different categories of melanoma. However, the molecular mechanisms of these genes in CoM cell evolution remain unexplored and await future investigations.

The TME, comprising stromal fibroblasts and immune constituents, exerts regulatory influence over the distant metastasis of diversified categories of melanoma^20^. However, the CoM TME has not been investigated at a single-cell resolution. Our study revealed the presence of CAFs exhibiting elevated expression of angiogenesis marker genes in DMT CoM. Previous studies have demonstrated that CAFs are the primary source of extracellular matrix components that contribute to tumor progression, which is consistent with our observations^34–36^. Moreover, CAFs also secrete a plethora of cytokines, chemokines, growth factors and exosomes, which may facilitate tumor progression and modulate treatment responses^37,38^.

VEGF has been shown to play an important immunosuppressive role in the TME and mediate T cell exclusion, which was required for T cell adhesion and extravasation^39^. In addition, tumor vasculature can overexpress the Fas ligand, which can induce apoptosis in transmigrating T cells expressing Fas and thus block T cell infiltration in CoMs, such as immune-excluded tumors^40^. On the other hand, previous studies have demonstrated that increased VEGF secretion decreases T cell effector function and trafficking to tumors^41^. Efforts to normalize the tumor vasculature to improve tumor tissue perfusion and immune function have been pursued by inhibiting the VEGF–receptor pathway in combination with immune checkpoint inhibitors in the treatment of metastatic renal cell carcinoma^42^ and hepatocellular carcinoma^43^.

Melanoma is a heterogeneous disease, with differences in therapeutic strategies at different sites and different stages^44^. Here, we depicted a distinct immune cell composition in DMT CoM characterized by exclusion of CD8^+^ T cells. Notably, checkpoint inhibitor therapy with anti-PD1 is one of the breakthroughs in the treatment of metastatic melanoma by activating CD8^+^ T cell response through various routes^45^. Previous studies have shown improved survival in patients with metastatic SKCM after anti-PD1 treatment^46^. However, therapeutic resistance was also observed in melanoma patients with PD1 blockade treatment^47^. We noticed a higher abundance of cancer-associated fibroblasts in the tumor microenvironment of DMT CoMs, correlated with enhanced angiogenic capacity and increased VEGFR expressions. Moreover, DMT CoMs exhibit immunosuppressive milieu, as evidenced by a reduced presence of effector/memory CD8^+^ T cells and an elevated abundance of naive CD8^+^ T cells. Given these key changes during CoM progression, we launched a clinical trial of VEGFR blockade combined with anti-PD1 therapy for distant metastatic CoM patients, which exhibited sufficient tumor-inhibitory efficacy.

Importantly, our data suggest that anti-angiogenesis therapy not only facilitates reduction of blood vessels, which facilitate malignant growth and metastasis, but also alters TME, thereby potentially intensifying the response of non-immunoreactive CoM^48^.

To enhance immune cell replenishment through reduction of blood vessels and the utilization of immune checkpoint blockade as a therapeutic target, a combination of VEGFR blockade (Apatinib) and anti-PD1 (Camrelizumab) therapy was employed. The combination was provided for distal metastatic CoM patients who were identified with increased expression of VEGFR. Compared to patients who did not receive systemic treatment following local excision, the aforementioned 2 patients who enrolled in the clinical trial and received anti-VEGFR and anti-PD1 therapy exhibited prolonged prognoses with increased median survival time by 6 times (3.91 months vs 23.71 months). However, the limited sample size hinders an in-depth analysis to evaluate the clinical potency of DMT CoM. Because CoM is a rare disease, it is necessary to fully address the clinical potential of anti-PD1/VEGFRi in long-term studies.

In conclusion, our work provides important insights into understanding the landscape of CoM and the characteristics of the stromal cells and immune cells in the TME, revealing the heterogeneity of CoM. Such complexity was revealed by the findings of CAF-induced boosted angiogenesis and tumor metastasis, and CD8^+^ T exclusion in DMT CoM. These observations motivated clinical decisions in CoM patients. The clinical trial outcomes provided novel clinical strategies for the treatment of DMT CoM.

## Materials and methods

### Samples collection and ethical approval

The study was conducted in accordance with Declaration of Helsinki and approved by the Institutional Review Board of the Shanghai Ninth People’s Hospital, Shanghai Jiao Tong University School of Medicine (SH9H-2019-T185-2). Validation cohort samples in this study were collected from histologically confirmed CoM patients (confirmed by the Department of Pathology, Shanghai Ninth People’s Hospital, Shanghai Jiao Tong University School of Medicine) between June 2018 to June 2022. Written informed consent was obtained from all patients. Human tumor specimens were obtained from CoM patients who had undergone wide excision with cryotherapy or exenteration as well as lymph node and distal metastatic site dissection. Eligible patients included in this study were age > 18 years with histologically confirmed conjunctival melanoma. Systemic metastasis cases were confirmed by imaging. Nonmalignant conjunctival samples were collected from patients who underwent local conjunctiectomy because of conjunctival nevus and eye traumas. All fresh tissues were dissected and enzymatically digested into single-cell suspensions.

### Study population and trial design

This clinical trial was performed at the Ninth People’s Hospital, Shanghai Jiao Tong University School of Medicine in Shanghai, China. Eligible patients were aged > 18 years, had histopathologically confirmed locally or generalized advanced CoM. Patients were enrolled between January 2021 to December 2022.

The trial followed the ethical guidelines of the Declaration of Helsinki and was approved by the Institutional Ethics Committee, Ninth People’s Hospital, Shanghai Jiao Tong University School of Medicine. Each patient provided signed informed consent before participating in this trial. An authorization of the release of the data was obtained from the Clinical Research Board of Ninth People’s Hospital. This trial was registered on Chinese Clinical Trial Registry (ChiCTR2100045061). In this study, Camrelizumab (200 mg, every 3 weeks, intravenous administration (i.v.)) and Apatinib (250 mg, once a day, oral administration (p.o.)) were free for patients.

### Tissue dissociation

ScRNA-seq experiment was performed by experimental personnel in the laboratory of NovelBio Bio-Pharm Technology Co., Ltd. Tissues were surgically removed and kept in MACS Tissue Storage Solution (Miltenyi Biotec) until processing. Tissue samples were first washed with phosphate-buffered saline (PBS), minced into small pieces (~1 mm^3^) on ice and enzymatically digested with 200 µL Enzyme H and 100 µL Enzyme R and 25 µL Enzyme A for 30 min at 37 °C, with agitation. After digestion, samples were sieved through a 70 µm cell strainer, and centrifuged at 300× *g* for 5 min. After supernatant was removed, pelleted cells were suspended in red blood cell lysis buffer (Miltenyi Biotec) to lyse red blood cells. After washing with PBS containing 0.04% BSA, cell pellets were re-suspended in PBS containing 0.04% BSA and re-filtered through a 35 μm cell strainer. Dissociated single cells were then stained for viability assessment using Countstar Fluorescence Cell Analyzer.

### ScRNA-seq

BD Rhapsody system was used for discovery cohort. Single cell capture was achieved by random distribution of a single-cell suspension across > 200,000 microwells through a limited dilution approach. Beads with oligonucleotide barcodes were added to saturation so that a bead was paired with a cell in a microwell. The cells were lysed in the microwell to hybridize mRNA molecules to barcoded capture oligos on the beads. Beads were collected into a single tube for reverse transcription and ExoI digestion. Upon cDNA synthesis, each cDNA molecule was tagged on the 5′ end (that is, the 3′ end of a mRNA transcript) with a unique molecular identifier (UMI) and cell barcode indicating its cell of origin. Whole transcriptome libraries were prepared using BD Rhapsody single-cell whole-transcriptome amplification (WTA) workflow including random priming and extension (RPE), RPE amplification PCR and WTA index PCR. The libraries were quantified using a High Sensitivity DNA chip (Agilent) on a Bioanalyzer 2200 and the Qubit High Sensitivity DNA assay (Thermo Fisher Scientific). Sequencing was performed by Illumina sequencer (Illumina, San Diego, CA) on a 150 bp paired-end run.

The scRNA-seq libraries of validation cohort 1 were generated using the 10X Genomics Chromium Controller Instrument and Chromium Single Cell 3’ V3 Reagent Kits (10X Genomics, Pleasanton, CA). Briefly, cells were concentrated to ~1000 cells/µL and loaded into each channel to generate single-cell Gel Bead-In-Emulsions (GEMs). After the RT step, GEMs were broken and barcoded–cDNA was purified and amplified. The amplified barcoded cDNA was fragmented, A-tailed, ligated with adapters and index PCR amplified. The final libraries were quantified using the Qubit High Sensitivity DNA assay (Thermo Fisher Scientific) and the size distribution of the libraries was determined using a High Sensitivity DNA chip on a Bioanalyzer 2200 (Agilent). All libraries were sequenced by Illumina sequencer (Illumina, San Diego, CA) on a 150 bp paired-end run.

### ScRNA-seq data analysis

ScRNA-seq data analysis was performed by NovelBio Bio-Pharm Technology Co., Ltd. with NovelBrain Cloud Analysis Platform. We applied fastp with default parameter filtering adapter sequence and removed low-quality reads to achieve the clean data^49^. UMI tools were applied for Single Cell Transcriptome Analysis to identify the cell barcode whitelist^50^. The UMI-based clean data were mapped to human genome (Ensemble version 91) utilizing STAR mapping with customized parameter from UMI-tools standard pipeline to obtain the UMIs counts of each sample^51^. Cells contained over 200 expressed genes and mitochondria UMI rate below 40% passed the cell quality filtering and mitochondria genes were removed in the expression table. Seurat package (version: 3.1.4, https://satijalab.org/seurat/) was used for cell normalization and regression based on the expression table according to the UMI counts of each sample and percent of mitochondrion rate to obtain the scaled data. PCA was constructed based on the scaled data with top 2000 high variable genes and top 10 principals were used for tSNE construction and UMAP construction.

Utilizing graph-based cluster method (resolution = 0.8), we acquired unsupervised cell cluster result based on the PCA top 10 principals and calculated the marker genes by FindAllMarkers function with wilcox rank sum test algorithm under following criteria: (1) lnFC > 0.25; (2) *P* < 0.05; (3) min.pct > 0.1. In order to identify the cell type detailed, the clusters of same cell type were selected for re-tSNE analysis, graph-based clustering and marker analysis.

### Pseudotime analysis

We applied the Single-Cell Trajectories analysis utilizing Monocle2 (http://cole-trapnell-lab.github.io/monocle-release) using DDR-Tree and default parameter. Before Monocle analysis, we selected marker genes of the Seurat clustering result and raw expression counts of the cell passed filtering. Based on pseudotime analysis, branch expression analysis modeling (BEAM Analysis) was applied for branch fate determination gene analysis.

### Cell communication analysis

To enable a systematic analysis of cell–cell communication molecules, we applied cell communication analysis based on the CellPhoneDB, a public repository of ligands, receptors and their interactions^52^. Membrane secreted and peripheral proteins of the cluster of different time point were annotated. Significant mean and Cell Communication significance (*P* < 0.05) was calculated based on the interaction and the normalized cell matrix achieved by Seurat Normalization.

### Differential gene expression analysis

To identify differentially expressed genes among samples, the function FindMarkers with wilcox rank sum test algorithm was used under following criteria: (1) lnFC > 0.25; (2) *P* < 0.05; (3) min.pct > 0.1.

### CNV estimation

Cells defined as endothelial cells and fibroblasts were used as reference to identify somatic CNVs with the R package infercnv (v0.8.2). We scored each cell for the extent of CNV signal, defined as the mean of squares of CNV values across the genome. Putative malignant cells were then defined as those with CNV signal above 0.05 and CNV correlation above 0.5.

### IF staining assay

Deparaffinized and rehydrated tissue samples or cells seeded on glass slides were fixed with 4% formaldehyde for 30 min, blocked with 5% normal goat serum (Vector) for 1 h, and permeabilized with 0.5% Triton X-100 for 15 min. Thereafter, they were incubated with the primary antibodies at 4 °C overnight and the corresponding secondary antibodies for 1 h at room temperature. The nuclei were stained with DAPI (Sigma-Aldrich) for 30 min at room temperature. Finally, IF images were taken with a ZEISS Axio Scope A1 upright microscope. The expression of target protein was measured by ImageJ 1.52 software.

### Cell culture

CAFs and myofibroblasts were derived from fresh surgically resected normal and CoM tumor tissues. An in-house pathologist evaluated initial pathologic diagnoses of conjunctival melanoma. The tissues were minced, and the fibroblasts were isolated by differential trypsinization. After adherent fibroblasts were incubated for 5–8 days until 80% confluence, the mixed fibroblasts were labeled by FAP and sorted by FACS to enrich FAP-positive CAFs. All procedures performed in this study involving human participants followed the ethical standards in Shanghai Ninth People’s Hospital, Shanghai Jiao Tong University School of Medicine and the 1964 Declaration of Helsinki and its later amendments or comparable ethical standards. Cells were incubated in a humidified incubator at 37 °C, supplied with 5% CO_2_. All of the cell lines were free of mycoplasma and pathogenic murine viruses.

### FACS

Briefly, tumors tissues were extracted and minced. Tumor cells were trypsinized and resuspended as single-cell suspensions on ice before sorting. Cells were resuspended in the staining buffer and stained with anti-FAP on ice for 30 min, and subsequently isolated on MoFlow XDP (Beckman Coulter, USA). These isolated FAP-positive fibroblasts were designated “CAFs”, and the remained cells were designated “myofibroblasts”.

### Flow cytometry analysis

Cells were stained with fluorochrome-conjugated antibodies. The antibodies used are detailed in Supplementary Table S11. The stained cells were then analyzed using a cytometer (BD FACSCalibur, BD Biosciences, CA).

### HUVEC tube formation assay

Twenty-four-well plates were precoated with 150 μL precooled Matrigel (Corning, 354234) per well and polymerized at 37 °C for 30 min. HUVECs (1 × 10^4^ cells) suspended in 200 μL of conditional medium were seeded and co-cultured with myofibroblasts and CAFs into each well and further cultured for 4 h. Then, the fields of tube structure were randomly chosen and photographed for quantification.

### Tissue processing for LCM

FFPE tissue sections were cut (8 μm), collected, air dried and heated at 65 °C for 60 min to facilitate better adhesion. Next, sections were deparaffinized, rehydratrated and loaded wet as following: 2 × 2 min xylene, 2 × 1 min 100% EtOH, 95% EtOH, 85% EtOH, 75% EtOH, 50% EtOH, and ddH20, respectively. The tissue sections then were stained with H&E as following. Each slide was processed with hematoxylin, bluing buffer and eosin for 7 min, 2 min and 40 s respectively. And the residual buffer used in the last step should be washed with water. H&E-stained tissue sections were then incubated at 37 °C for 5 min and visualized on digital slide scanner SLIDEVIEW VS200 (OLYMPUS) at 20× magnification.

LCM procedure was completed on Laser-Capture Microdissection System PALM (Zeiss). The LCM system was turned on and kept on for 15 min to stabilize the laser energy. And microscope and laser settings were set up as follows: zoom: 20×; cut energy: 39; focus: 62; catapulting energy: 64; focus: 70; cycle number: 1; cut speed: 100. The dried H&E-stained slide was putted onto the slide adapter of LCM microscope. The regions of interest containing a specific cell type in the section was marked with LCM marker pen and microdissected using above mentioned settings and collected with microtubes (Zeiss, 415190-9201-000). The stromal regions of CoMs were isolated through the utilization of LCM, a method that was subsequently validated by the pathologist affiliated with the Department of Pathology at Shanghai Ninth People’s Hospital. The microdissected samples were stored at –80 °C or digested for proteomic profiling.

### Sample preparation and MS analysis

The microdissected samples were resuspended with 5 μL lysis buffer and were sonicated for 3 min using a contactless high intensity ultrasonic processor (Scientz) and then were incubated at 95 °C for 5 min. Protein digestion was performed overnight at 37 °C after adding 1 μL of 50 ng trypsin. The peptides were desalted using C18 Zip Tips according to the manufacturer’s instructions and then dried for further MS analysis.

Spatial proteomic analysis was performed by PTM biolabs. The tryptic peptides were dissolved in solvent A, directly loaded onto a home-made reversed-phase analytical column (25-cm length, 100 μm i.d.). The mobile phase consisted of solvent A (0.1% formic acid, 2% acetonitrile/in water) and solvent B (0.1% formic acid, 90% acetonitrile/in water). Peptides were separated with the following gradient: 0–1.6 min, 4%–22.5%B;1.6–2.0 min, 22.5%–35%B;2.0–2.1 min, 35.0%–35.1%B;2.1–2.3 min, 35.1%B;2.3–9.2 min, 35.1%–35.2%B;9.2–9.6 min, 35.2%–55.0%B;9.6–10.1 min, 55.0%–99.0%B;10.1–12.0 min, 99%B, and all at a constant flow rate of 200 nL/min on a Vanquish Neo UPLC system (ThermoFisher Scientific). The separated peptides were analyzed in Orbitrap Astral with a nano-electrospray ion source. The electrospray voltage applied was 1900 V. Precursors were analyzed at the Orbitrap detector, and the fragments were analyzed at the Astral detector. The full MS scan resolution was set to 240,000 for a scan range of 400–800 m/z. The MS/MS scan was fixed first mass as 150.0 m/z at a resolution of 80,000. The HCD fragmentation was performed at a normalized collision energy of 25%. Automatic gain control target was set at 800%, with a maximum injection time of 15 ms.

### Statistical analysis

Statistical analysis was performed using GraphPad Prism 9.2. Comparisons were assessed using Student’s *t*-test or one-way ANOVA where appropriate. Data are presented as means ± SD. *P* < 0.05 was considered statistically significant.

### Supplementary information

Supplementary information

## Data Availability

ScRNA-seq data of discovery cohort have been deposited on Gene Expression Omnibus (GEO) platform under the accession code “GSE217707”. ScRNA-seq data of validation cohort 1 have been deposited on National Omics Data Encyclopedia platform (NODE, https://www.biosino.org/node/) under the accession code “OEZ01468 ~ OEZ01474”.
