# Supplementary material for: Single-cell sequencing depicts tumor architecture and empowers clinical decision in metastatic conjunctival melanoma
Source: Cell Discov. 2024 Jun 11;10:63. doi: 10.1038/s41421-024-00683-y (PMC11166926; doi:10.1038/s41421-024-00683-y)
Supplement: Supplementary file 1 — Supplementary information [file 41421_2024_683_MOESM1_ESM.pdf]

Supplementary Information for

Single-cell sequencing depicts tumor architecture and empowers clinical decision in metastatic conjunctival melanoma

Hanhan Shi[#1,2,3], Hao Tian[#1,2,3], Tianyu Zhu[#1,2,3], Qili Liao[#1,2,3], Chang Liu[1,2,3], Peng Yuan[4], Yongyun Li[1,2,3], Jie Yang[1,2,3], Chunyan Zong[1,2,3], Shichong Jia[5], Jing Ruan[1,2,3], Shengfang Ge[1,2,3], Renbing Jia*[1,2,3], Peiwei Chai*[1,2,3], Shiqiong Xu*[1,2,3], Xianqun Fan*[1,2,3]

[1]Department of Ophthalmology, Shanghai Ninth People's Hospital, Shanghai Jiao Tong University School of Medicine, Shanghai, China. [2]Shanghai Key Laboratory of Orbital Diseases and Ocular Oncology, Shanghai, China. [3]Center for Basic Medical Research and Innovation in Visual System Diseases of Ministry of Education, Shanghai Jiao Tong University School of Medicine, Shanghai, China. [4]State Key Laboratory of Molecular Biology, Shanghai Key Laboratory of Molecular Andrology, CAS Center for Excellence in Molecular Cell Science, Shanghai Institute of Biochemistry and Cell Biology, Chinese Academy of Sciences-University of Chinese Academy of Sciences, Shanghai, China. [5]Tianjin Eye Hospital, Tianjin Key Lab of Ophthalmology and Visual Science, Nankai University Affiliated Eye Hospital, Tianjin Eye Institute, Tianjin, China.

These authors contributed equally: Hanhan Shi, Hao Tian, Tianyu Zhu, Qili Liao

Correspondence: Xianqun Fan (fanxq@sjtu.edu.cn), Shiqiong Xu (115033@sh9hospital.org.cn), Peiwei Chai (chaipeiwei123@sjtu.edu.cn), Renbing Jia (renbingjia@sjtu.edu.cn)

List of Supplementary Materials

Materials and Methods

Fig. S1 to S8 for multiple supplementary figures

Table S1 to S11 for multiple supplementary tables

Fig. S1

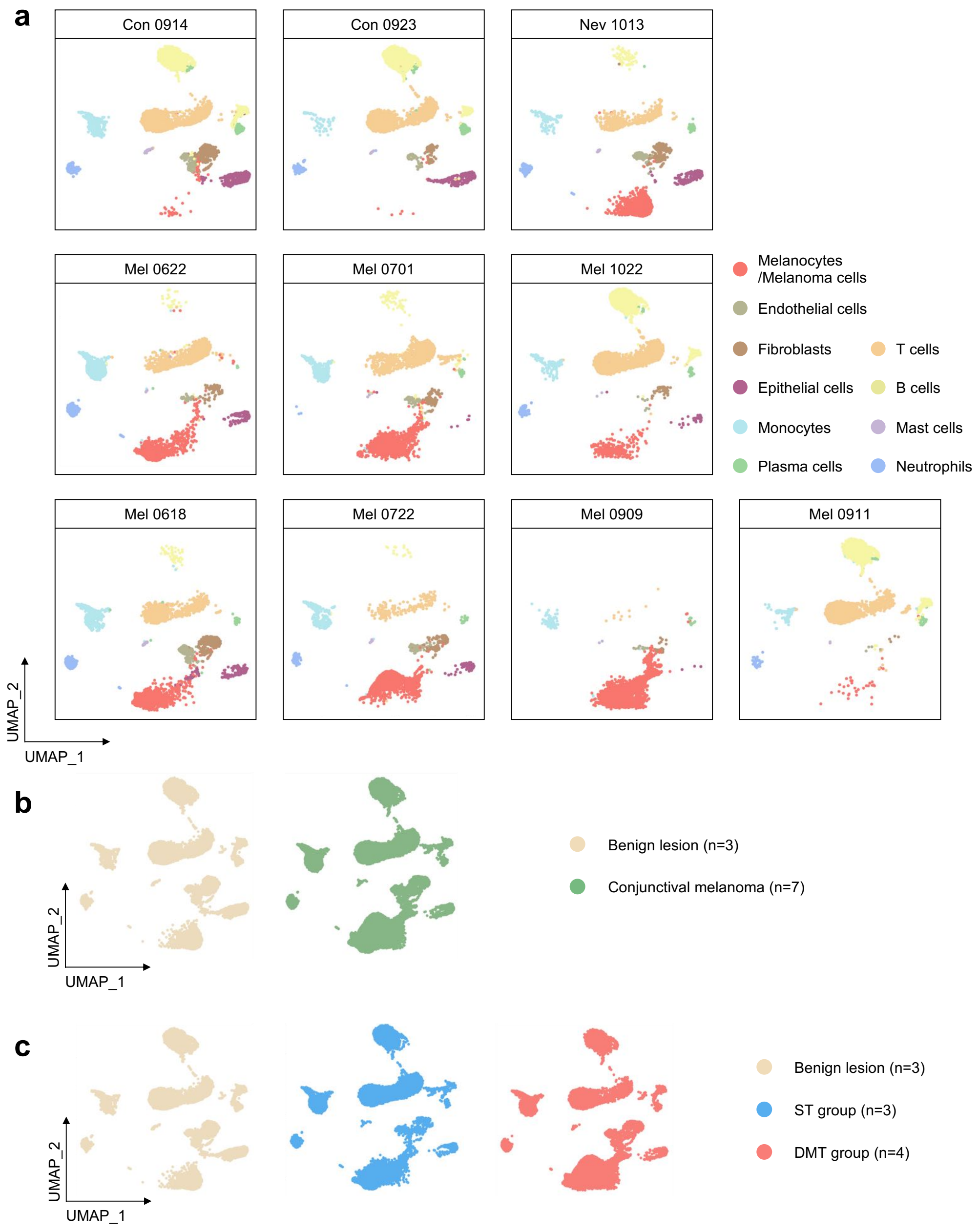

Supplementary Figure S1. UMAP plot illustrating diverse cell types from different samples.

(a) UMAP plot showing diverse cell distribution colored by cell annotations.

(b-c) UMAP plot showing diverse cell distribution by color, benign lesion or melanoma origin (b, benign lesion, white; melanoma, green), and ST or DMT origin (c, ST group, blue; DMT group, red).

Fig. S2

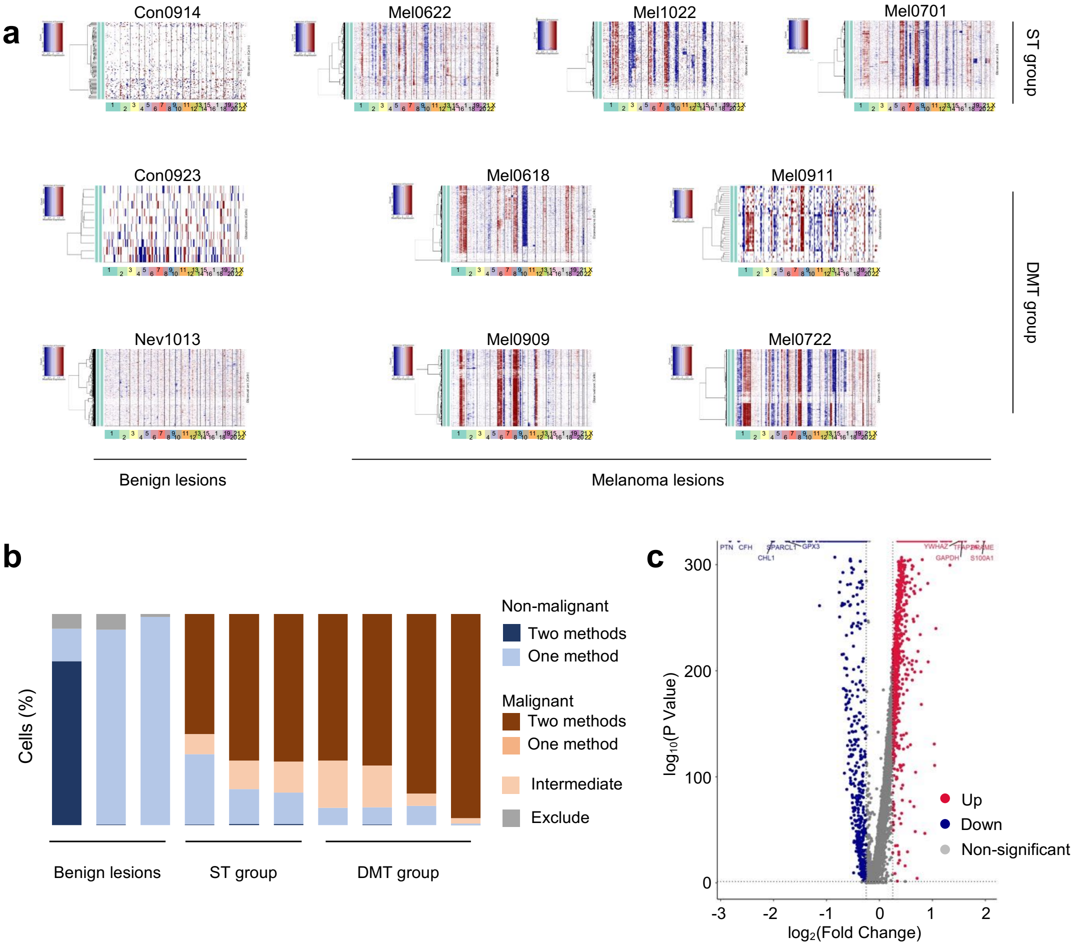

Supplementary Figure S2. Two complementary methods confidently distinguished melanomas and nonmalignant melanocytes.

(a) Chromosomal copy-number variations (CNVs) in each single cell from different samples were inferred according to the averaged expression profiles across chromosomal intervals.

(b) Stacked bar plots of melanocytes/melanoma cells show percent of malignant (red) and non-malignant (blue) cells, as classified by one (light color) or two (dark color) independent methods: CNVs and marker scoring.

(c) Volcano plots of differentially expressed genes between melanocytes and malignant melanoma cells. The X- axis represents log fold changes. The Y-axis represents log p values. The blue points denote the significantly downregulated genes, and the red points denote the significantly upregulated genes.

Fig. S3

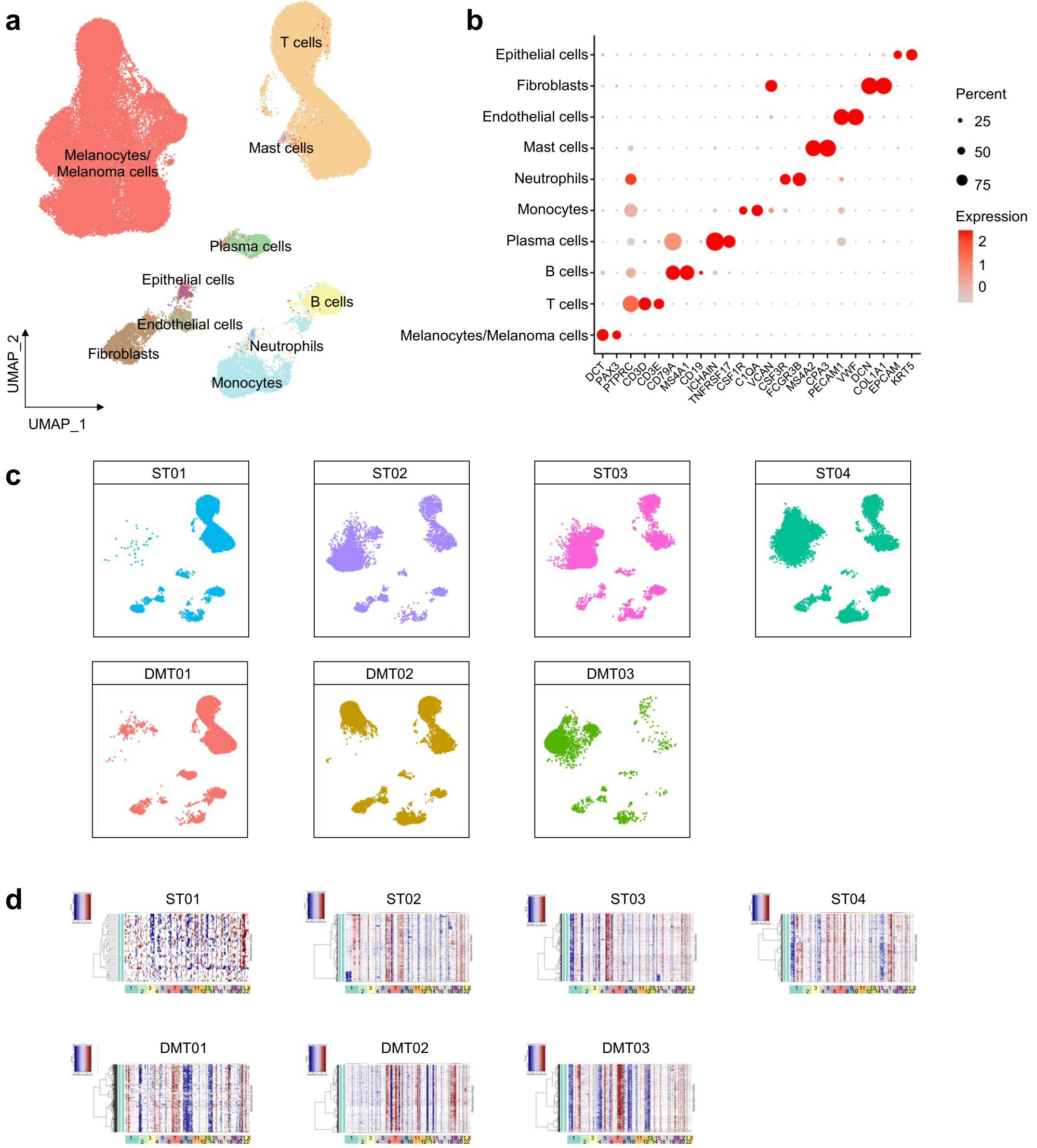

Supplementary Figure S3. UMAP plot illustrating diverse cell types from different samples in validation cohort 1.

(a) UMAP plot illustrating the annotation and different colors for diverse cell types in primary ST (n=4) and DMT (n=3) CoM.

(b) Dot plot showing the expression levels of the specific cell marker genes in different cell types. The size of the dot suggests the proportion of cells expressing the marker genes. The spectrum of colors indicates the mean expression levels of the marker genes.

(c) UMAP plot showing diverse cell distribution colored by patient origin.

(d) Chromosomal copy-number variations (CNVs) in each single cell from different samples were inferred according to the averaged expression profiles across chromosomal intervals in validation cohort 1.

Fig. S4

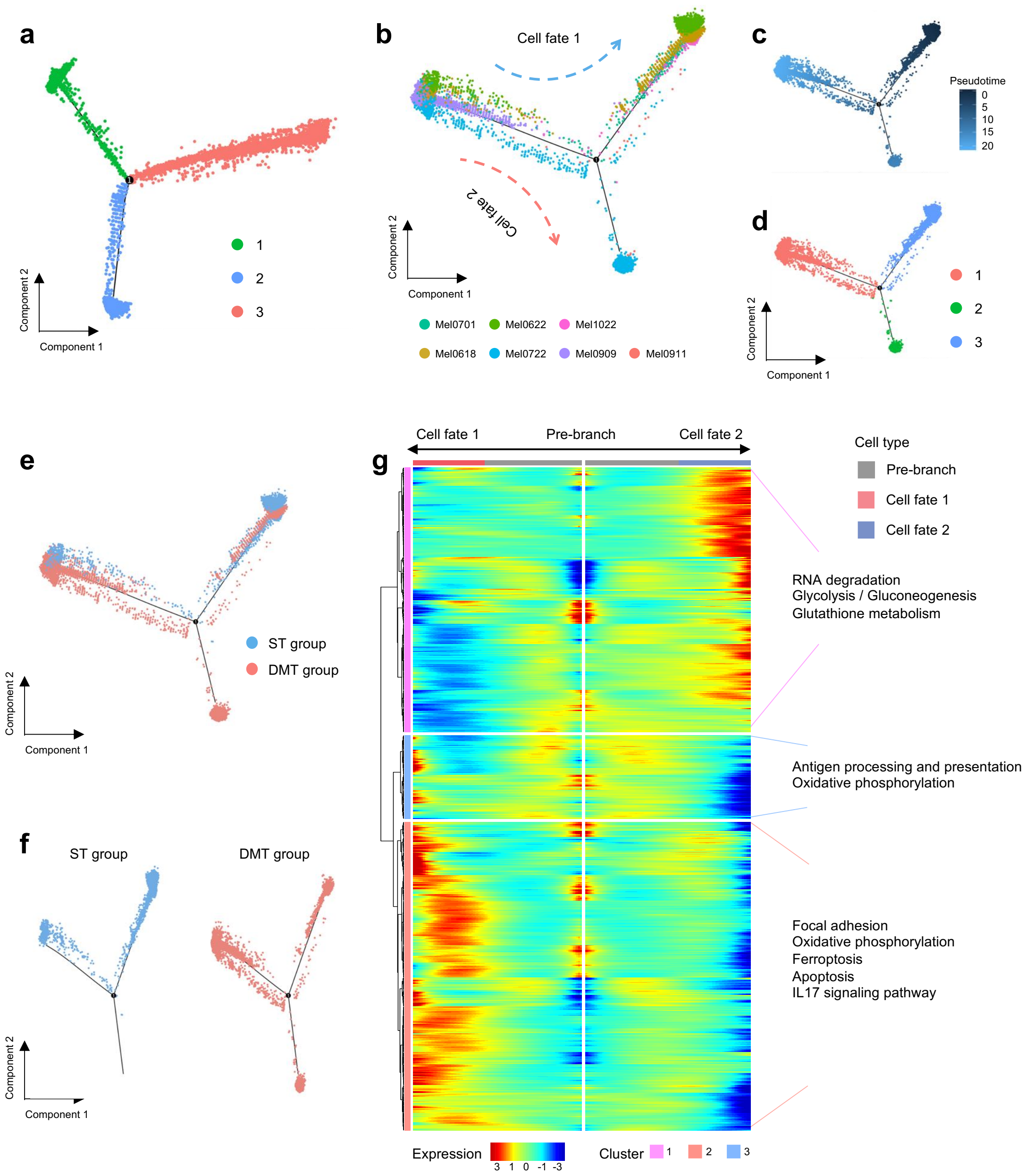

Supplementary Figure S4. Transcriptional trajectory analysis of CoM.

(a) Transcriptional trajectory of different process colored by clusters.

(b) Pseudotime state of all melanoma cells from both the ST and DMT groups colored by patient origin.

(c-f) Transcriptional trajectory analysis showing different process of melanoma cells by color, states (c), clusters (d), ST or DMT group origin (e-f).

(g) Heatmap shows upregulated or downregulated genes in the differentiation process. The differentially expressed genes (rows) along the pseudotime (columns) were hierarchically clustered into three subclusters. The representative enriched pathways of each subcluster are provided.

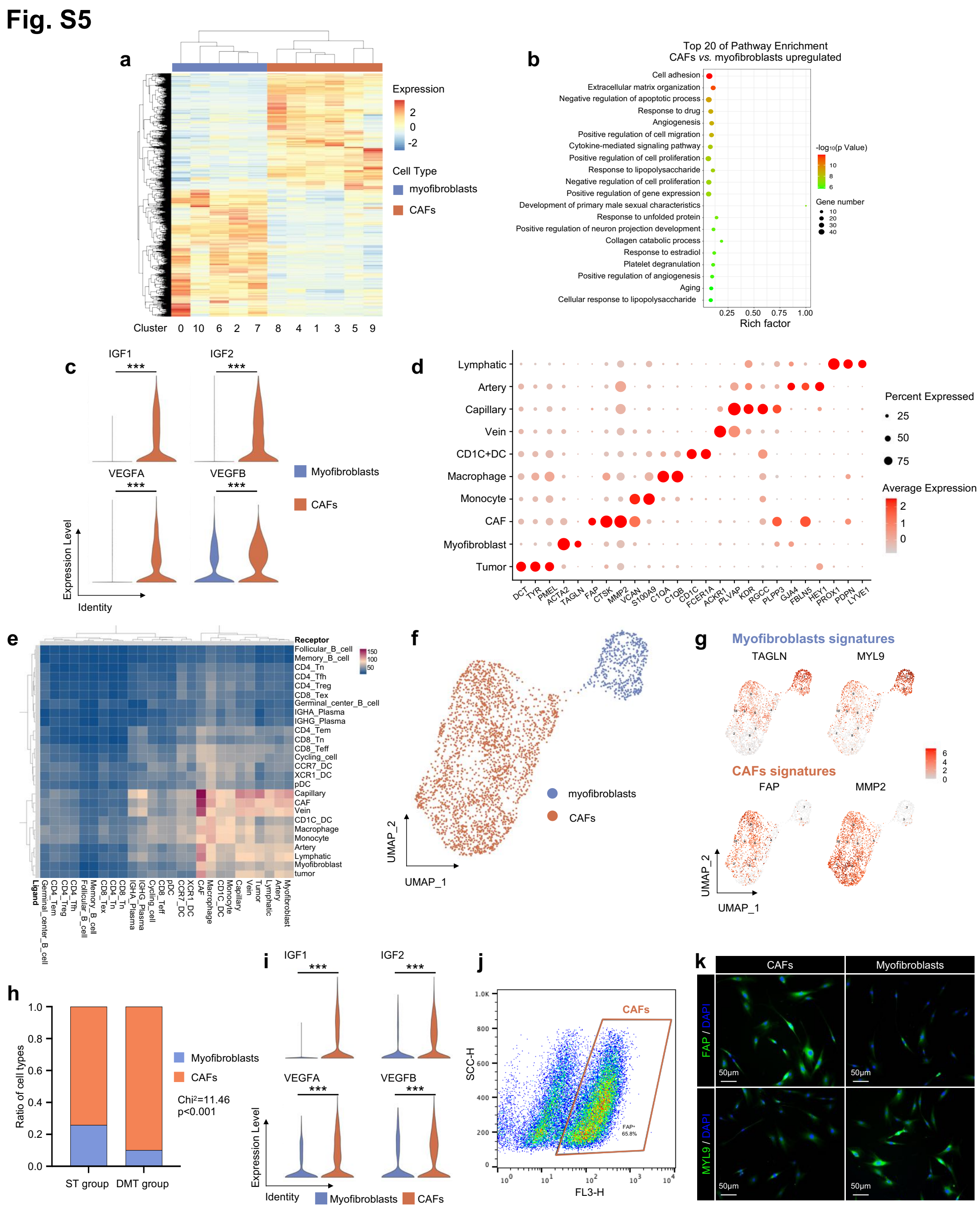

Supplementary Figure S5. Molecular characteristics of stromal cells in discovery cohort and validation cohort 1 of CoM samples.

(a) Heatmap plot showing the extremely different expression in CAFs (red) and myofibroblasts (blue), upregulated genes in red and down regulated genes in blue.

(b) Bubble plots of upregulated expressed genes between CAFs and myofibroblasts enriched pathways. The top 20 processes were displayed.

(c) Violin plots indicating the expression of angiogenesis-associated genes in CAFs (orange) and myofibroblasts (blue) in discovery cohort.

(d) Dot plot showing the expression levels of the specific cell marker genes in different cell types. The size of the dot suggests the proportion of cells expressing the marker genes. The spectrum of colors indicates the mean expression levels of the marker genes.

(e) Cell phone analysis showing the interaction of melanoma cells, CAFs and the immune microenvironment. The spectrum of colors indicates the correlation between two cell types.

(f) UMAP plot showing the subtypes of CAFs derived from ST and DMT patients in validation cohort 1, colored by cell type, myofibroblasts in blue and CAFs in orange.

(g) UMAP plot in validation cohort 1 was stained by the expression of marker genes of myofibroblasts (*TAGLN*, *MYL9*), and CAFs (*FAP*, *MMP2*).

(h) Bar plots showing the proportion of CAFs (orange) and myofibroblasts (blue) in ST (left) and DMT (right) samples. Cell type annotations are provided in the figure (right). The difference was statistically significant. All statistical analyses are Chi-square tests.

(i) Violin plots indicating the expression of angiogenesis-associated genes in CAFs (orange) and myofibroblasts (blue) in validation cohort 1.

(j) Isolation of CAF subtypes (FAP positive) from CoM tumor tissues via FACS. The clinical distal metastatic tissues were derived from Mel0618.

(k) IF staining with FAP (up) and MYL9 (bottom) antibody in CAFs (left) and myofibroblast (right). Scale bars, 50 μm.

Fig. S6

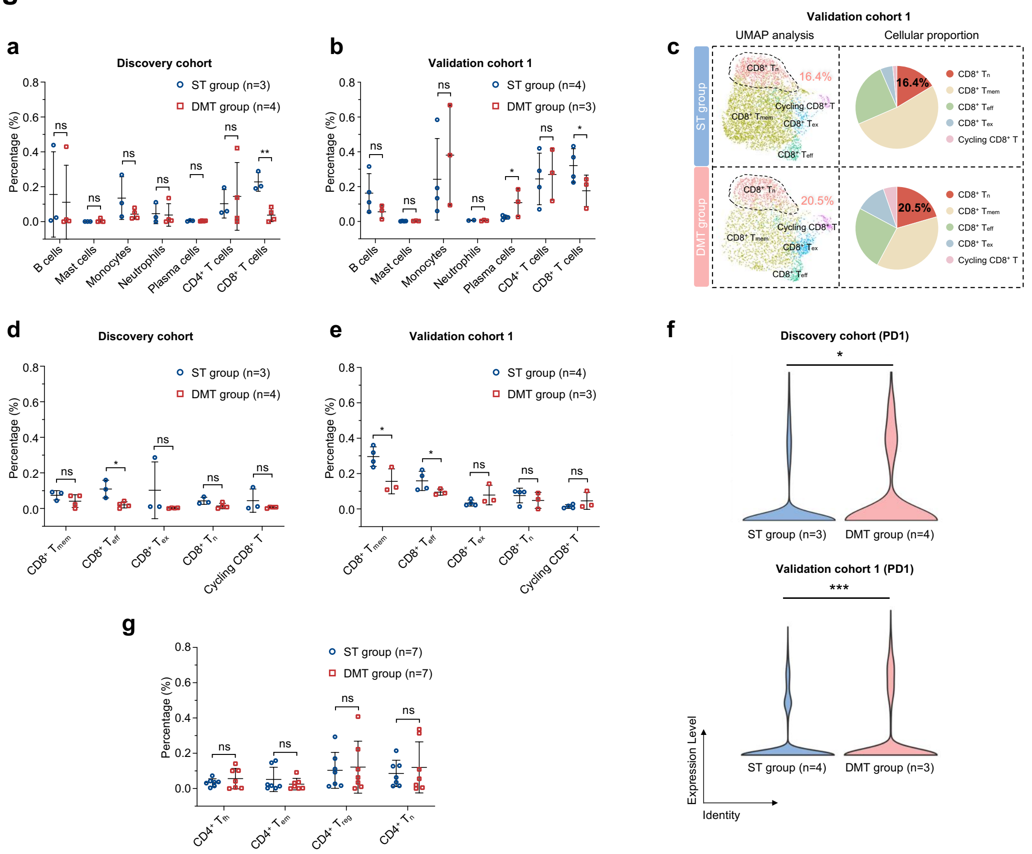

Supplementary Figure S6. Molecular characteristics of immune cells in discovery cohort and validation cohort 1 of CoM samples.

(a) Proportion of each immune cell subset in the ST group (blue) and DMT group (red) from discovery cohort. Two-tailed unpaired Student's t-test.

(b) Proportion of each immune cell subset in the ST group (blue) and DMT group (red) from validation cohort 1. One-tailed unpaired Student's t-test.

(c) The proportions of specific subsets of $CD8^+$ T cells between the ST group and DMT group were analyzed (left). Pie charts showing the proportion of $CD8^+$ T_n cells (red) in ST and DMT groups (right) from validation cohort 1.

(d) The infiltration percentage of specific subsets of $CD8^+$ T cells between the ST group (blue, n=3) and DMT group (red, n=4) were analyzed in discovery cohort. Two-tailed

unpaired Student's t-test.

(e) The infiltration percentage of specific subsets of $CD8^+$ T cells between the ST group (blue, n=4) and DMT group (red, n=3) were analyzed in validation cohort 1. One-tailed unpaired Student's t-test.

(f) Violin plots indicating the expression of clinical therapy target (PD1) in $CD8^+$ T_{eff} cells from ST group (blue) and DMT group (red) in discovery cohort (left) and validation cohort 1 (right), respectively.

(g) Dot plots showing the infiltration level of $CD4^+$ T cells in the ST (blue, n=7) and DMT group (red, n=7) from both discovery cohort and validation cohort 1. Two-tailed unpaired Student's t-test.

Fig. S7

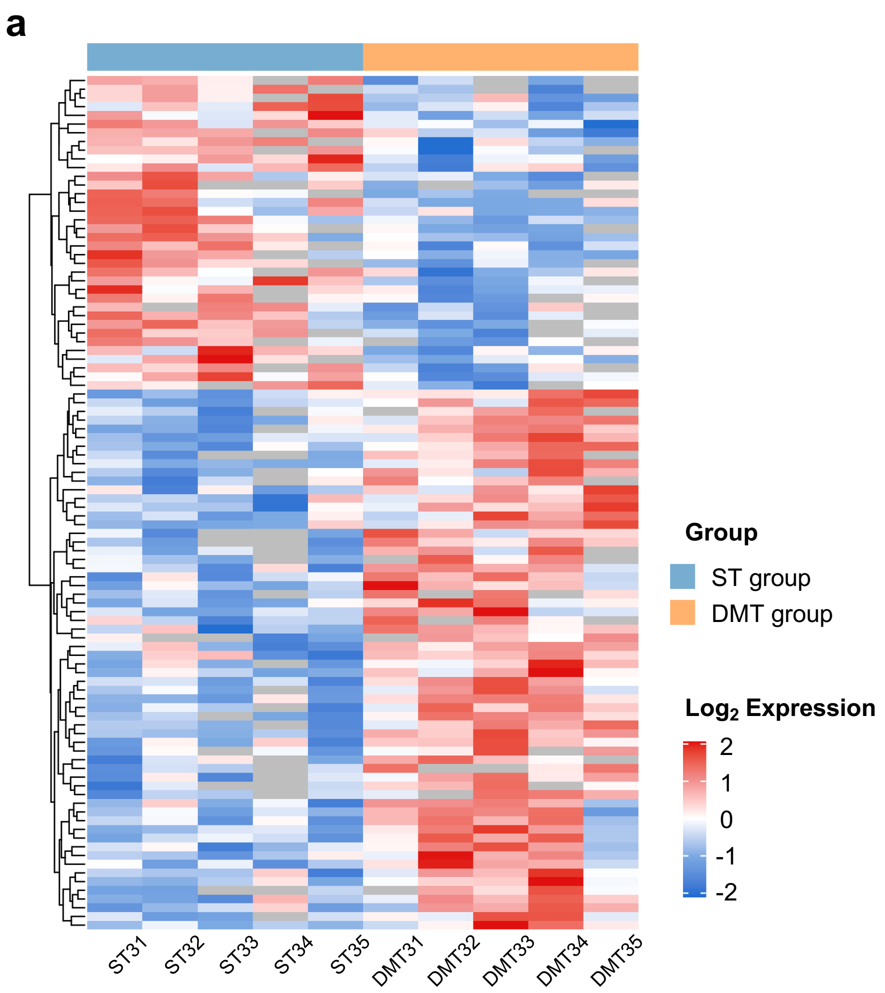

Supplementary Figure S7. Different expressed proteins in ST and DMT CoMs.

(a) Heatmap plot showing the different expression in ST group (blue) and DMT group (orange), upregulated proteins in red and down regulated proteins in blue.

Fig. S8

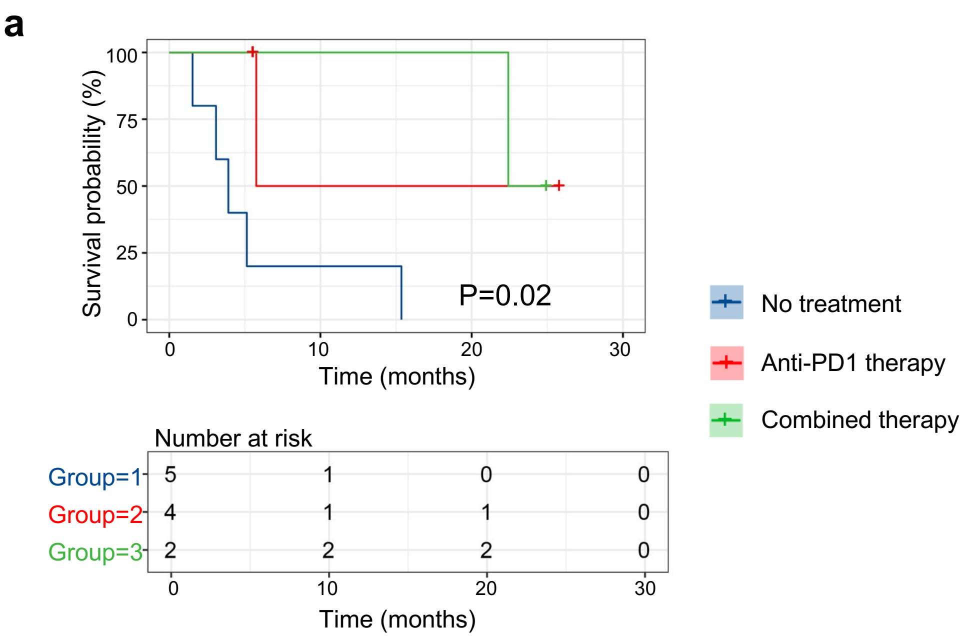

Supplementary Figure S8. Clinical efficacy of VEGFR blockade combined with anti-PD1 therapy.

(a) Survival analysis of distant metastatic CoM patients received different treatment.

Supplementary Table S1. Patient and tumor characteristics at presentation for single cell RNA sequencing

Patient	Patient 01	Patient 02	Patient 03	Patient 04	Patient 05	Patient 06	Patient 07	Patient 08	Patient 09	Patient 10
Age	56	48	47	68	60	66	60	51	61	56
Sex	Male	Male	Male	Female	Male	Female	Male	Male	Male	Female
Samples name	Con0914	Con0923	Nev1013	Mel0622	Mel0701	Mel1022	Mel0911	Mel0618	Mel0722	Mel0909
Tissues types	Conjunctiva	Conjunctiva	Conjunctiva	Melanoma	Melanoma	Melanoma	Melanoma	Melanoma	Melanoma	Melanoma
Tumor location				Bulbar conjunctiva	Orbit	Palpebra	Eyelid	Eyelid	Bulbar conjunctiva & Palpebra	Eyelid
Tumor thickness				8	11	4.2	3.9	0.8	3.5	4.5
Ulceration				Absent	Present	Absent	Present	Absent	Present	Absent
TIL				Brisk	Absent	Absent	Brisk	Brisk	Brisk	Brisk
Regression				Absent	Absent	Absent	Absent	Absent	Absent	Absent
Mitotic rate				6	6	7	12	7	2	13
Perineural invasion				Absent	Absent	Absent	Absent	Absent	Absent	Absent
Vascular invasion				Absent	Absent	Absent	Absent	Absent	Absent	Absent
Microscopic satellitosis				Absent	Absent	Absent	Absent	Absent	Absent	Absent
Clinical T category at presentation				T3c	T3b	T2d	T3b	T3c	T3d	T3c
N category				0	0	0	1	0	1	1
M category				0	0	0	1	0	1	0
Sites of distant metastasis				0	0	0	Liver	Liver, Lumbar	Liver	Liver, Bone, Brain

Pathological T category at presentation				T3c	T3b	T2b	T3b	T3c	T3d	T3c
Origin				Nevus	De Novo	Nevus	PAM	Nevus	De Novo	De Novo
Driver mutation				NF1 c.5812+2T>G	NRAS Q61R	BRAF V600E	NA	BRAF V600E	NRAS G12A	NRAS Q61R
Follow-up time (mo)				31.30	43.00	27.29	10.58	13.68	15.35	11.74
Prior treatments				NA	NA	Resection	NA	NA	Resection	NA
Presence of immunological disorders				NA	NA	NA	NA	NA	NA	NA
History of cancer				NA	NA	NA	NA	NA	NA	NA
Group name	Benign lesions	Benign lesions	Benign lesions	Stable tumor	Stable tumor	Stable tumor	Distant metastatic tumor	Distant metastatic tumor	Distant metastatic tumor	Distant metastatic tumor
Prognosis				Stable	Stable	Stable	Dead	Dead	Dead	Dead

Supplementary Table S2. Conjunctival melanoma Patient and Tumor Characteristics in validation cohort.

	No
Gender	
Male	15
Female	22
Age Mean (range)	58.0 (31-80) years
≤60 years	21
>60 years	16
Tumor origin	
De novo	17
Nevus	3
PAM	17
Tumor location	
Bulbar conjunctiva	28
Limbus	6
Cornea	5
Palpebra	30
Fornix	6
Caruncle	4
Eyelid	18
Orbit	9
Tumor Thickness	
≤4 mm	20
>4 mm	17
Ulceration	
Absent	18
Present	19
TIL	
Absent	7
Nonbrisk	24
Brisk	6
Mitotic Rate	
≤10	22
11~20	7
≥21	8
Regression	

Absent	31
Present	6
Vertical growth plate	
Absent	3
Present	34
Perineural invasion	
Absent	31
Present	6
Vascular invasion	
Absent	25
Present	12
Microscopic satellitosis	
Absent	30
Present	7
Clinical T category at presentation	
T1	0
T2	18
T3	19
T4	0
N category at presentation	
N0	32
N1	5
M category at presentation	
M0	34
M1	3

M=distant metastasis; N=regional lymph node; T=tumor; TIL= tumor-infiltrating lymphocyte

Supplementary Table S3. Cell number of assigned cell clusters in CoM and Benign conjunctival samples

Clusters	Total	Benign conjunctiva	CoM	Cell type	Main markers
0	7081	1950	5131	T cell	CD3D, CD3E
1	7306	2785	4521	B cell	CD79A, MS4A1, CD19
2	5085	1449	3636	T cell	CD3D, CD3E
3	5482	86	5396	Melanocyte/melanoma	DCT, TYR, PAX3, PMEL
4	3395	50	3345	Melanocyte/melanoma	DCT, TYR, PAX3, PMEL
5	3876	2737	1139	Melanocyte/melanoma	DCT, TYR, PAX3, PMEL
6	1702	174	1528	Melanocyte/melanoma	DCT, TYR, PAX3, PMEL
7	2336	318	2018	Monocyte	LYZ, CSF1R, C1QA, VCAN, FLT3, CD1C
8	1858	1201	657	Fibroblast	DCN, COL1A1, COL1A2
9	1912	1443	469	Epithelial	EPCAM, KRT13
10	672	524	148	Plasma cell	JCHAIN, TNFRSF17
11	1626	300	1326	Neutrophil	CSF3R, S100A9, FCGR3B
12	798	307	491	Endothelial	PECAM1, VWF, CLDN5
13	616	273	343	Fibroblast	DCN, COL1A1, COL1A2
14	860	586	274	B cell	CD79A, MS4A1, CD19
15	689	177	512	Monocytic	LYZ, CSF1R, C1QA, VCAN, FLT3, CD1C
16	305	2	303	Melanocyte/melanoma	DCT, TYR, PAX3, PMEL
17	379	61	318	T cell	CD3D, CD3E
18	422	348	74	Endothelial	PECAM1, VWF, CLDN5
19	255	2	253	Melanocyte/melanoma	DCT, TYR, PAX3, PMEL
20	239	119	120	Mast cell	MS4A2, CPA3
21	105	34	71	B cell	CD79A, MS4A1, CD19
22	18	13	5	Neutrophil	CSF3R, S100A9, FCGR3B

Supplementary Table S4. Pseudotime statistics

Cluster	Melanocytes	Melanoma cells
1	4	4192
2	2976	529
3	61	7243

Supplementary Table S5. Differentially expressed genes along trajectory

Gene								
LINC02367	TBC1D7	YWHAH	HSPA1A	TNFAIP3	LAMP2	DYNLRB1	ANLN	SNX10
CRYAB	PYGB	WBP2	SRPRA	CXCL1	PRSS23	GAS2L3	WISP2	CFL1
A2M	CLTC	AP3S1	SYPL1	LDLR	SGCB	CYGB	HLA-DOA	HMG20B
GSTP1	GPAT4	VKORC1	CAMK2N1	HLA-DQA2	PLP2	CAPNS1	TRPM8	NPL
TFAP2A	MAPK1	ALDOA	PSME2	CXCL2	RHOC	RNF187	NUSAP1	TRAPPC1
TSPAN10	PCNA	MSANTD3	UBB	ENTHD1	FAM69C	MBP	ATP6V1D	TSPO
GPR143	C1QB	COPE	TGIF1	NCOA7	GJC1	SMIM14	ERO1A	ORAI1
PAG1	CCL18	UBL3	RHOA	OR7A5	CDC42EP3	CD63	MFSD12	VAMP8
SPARC	GDF15	STAT1	ATP5H	FRS2	PCMT1	STXBP1	CITED1	NDUFB1
LINC00518	MSC	IFT81	H2AFV	TYROBP	QPCT	PMEL	RBPMS2	RUNX1
CALU	TRIB1	CAPN2	GPR137B	REEP6	MLANA	KIT	PMP22	CXCL10
F2R	ACSL3	SHC4	SHISA5	TIMP1	ENPP2	COX8A	LGALS3BP	KPNA2
LYST	PSEN2	AP1S2	TUBB2B	CENPX	HPS5	CPEB4	C5AR1	HSPA5
CDC42	CDK2	SNCA	LGALS3	TMEM50A	H2AFJ	LPGAT1	LAMTOR5	NES
RASSF2	TMED9	UBE2A	ECM1	H3F3B	NSL1	LDHA	SRPX	MCAM
PTP4A1	NQO1	FAM126A	PKM	IL1RAP	KCTD3	CHCHD2	TYRP1	BRD2
KREMEN1	PLAT	EMP3	DUSP14	HOPX	PPP2R5A	PARK7	HSPB1	CYR61
TYR	GRN	XG	ARHGDIA	HLA-DMB	NENF	POLE3	TXN	HSP90B1
CYP27A1	SIPA1L2	UQCR11	SEC61B	HLA-DPA1	APOD	NDUFB2	PFN1	PPIA
APOE	UBC	HPGD	ATP6V0D1	FAM129A	RAB11B	ARF1	TAGLN2	ATP6V1F
CTSB	ATP6AP1	TTYH2	GRB2	HLA-DQB1	GNAS	ATP5J2	ACTB	HLA-DMA
CTSL	VGF	CYSLTR2	C1QC	CXCL8	ATP6V1G1	HEXB	FTL	OAS2

PLA1A	SPP1	MXRA7	SERPINE1	HLA-DPB1	PPDPF	COX6B1	ATP5E	UPP1
MME	AKAP6	SLC3A2	PCSK2	CTSS	CPQ	VMP1	PGK1	PLEK
RNASE1	SAP18	EMP1	CNN2	SHISA2	SPAG9	ACTR3	MPV17	LYZ
CARD16	MFGE8	UBALD2	UFM1	OR7C1	GDI1	CNIH4	IGSF8	DAAM1
APOC1	NSG1	IFI6	EZR	ACTN1	PLSCR1	PLEKHB2	SERPINF1	ZDHHC20
ICAM1	DSTYK	RAB32	NFKBIA	B4GALT1	CST3	TRIB2	G6PC3	ATP6V0E1
WARS	MT2A	TUBA1C	MOSPD1	PLAUR	TCEAL3	PLP1	SLC39A6	SERPINH1
SERPINA1	BANCR	ORC5	MYL12A	BCL3	NUCB1	PTTG1IP	ATP1A1	TOP2A
HLA-DRB5	ANXA5	ABCB5	UBL5	MAOB	PRNP	ZFYVE16	ABL2	PTBP3
TAP2	FMN1	ATF3	RAC1	CD14	IFITM2	ACOT7	YWHAB	HLA-C
LAMP1	GMPR	DLL3	MAN1A2	RFK	SYNE2	PI15	CDKN1A	STOM
CTNND1	CKS2	POMP	STX3	ANXA1	CEACAM1	PACSIN2	CDK2AP2	IL13RA1
DDX5	SEMA6A	ERH	SOX13	CD74	PRKD3	AKAP9	SGK1	MAPKAP1
CTSD	H2AFZ	P4HB	BAX	ANXA2	TMEM30A	TMEM245	SYTL2	CCL5
C1S	SCIN	HNRNPA2B1	DUSP4	S100A6	S100A10	SPTBN1	TUBB2A	CAPG
JUN	CDH3	ATP6V0B	SERF2	SELENOK	PSMB1	TXNIP	IFI27	MDM2
HES1	ASPA	UVRAG	PALMD	SNX9	PSMB7	FYN	KCNN2	IER5
TNFRSF12A	MLPH	GPX4	CENPF	FCER1G	ANKRD28	RPS4Y1	VAV3	FN1
PICALM	RAB7A	ANOS1	TAF13	CCL4L2	SDC2	DDX3Y	NR4A3	MFSD14B
ARF6	BCAN	PHLDA2	MAGEC2	IL1B	PPP6C	ANP32B	IER2	ARPC2
KLF4	SLC1A4	GNG5	HLA-G	POLR2E	TSC22D3	MTURN	TOB1	RAB4B
FOSB	VEPH1	CDH1	RPH3A	EPB41L3	SRP9	ATP5L	CALR	ALYREF
ARPC3	EIF1	HLA-A	FDCSP	LIPA	PIK3R1	GSTO1	TSC22D1	DBI
MBNL2	CBX3	PRKAR1A	DUSP10	IL1RN	MYL12B	SLC35D2	CEBPB	GUK1
LIMA1	SMC6	CD151	HLA-DQA1	LCP1	ARL2	LEF1	EGR1	NFKBIZ

MATN2	C1orf21	RHBDD2	AK5	CCL3	TMSB10	S100B	CTNNB1	KDELR3
DNAJB1	OTUD7B	CYTH3	CHN1	CD53	COX7A2	DAZAP2	ZFAND5	IRF2BP2
RPN2	ARPC5	S100A11	FCRLA	MCF2L	ATP6V1B2	AES	TCEAL9	MYADM
RHOB	MGP	CSTB	RNF128	ITGB2	RAB27A	MAP4	JUND	KLF6
HSPA1B	FKBP1A	SMIM29	CTSZ	HLA-DRA	ATP6V1E1	CD99	HSPA8	NAPA
HLA-DRB1	DAD1	NDUFA4	CCL4	NTRK2	BIRC5	IFITM3	SNRPB	TMBIM6
PPP1R15A								

Supplementary Table S6. Pseudotime statistics

Cluster	ST group	DMT group
1	524	4750
2	2	2808
3	2456	1424

Supplementary Table S7. Cell number of assigned cell clusters of CAFs and myofibroblasts in each sample

Clusters	Total	Mel0618	Mel0622	Mel0701	Mel0722	Mel0909	Mel1022	Cell type	Main markers
0	257	48	3	0	3	1	0	Myofibroblasts	TAGLN, MYL9, TPM1, TPM2
1	240	80	5	1	9	2	3	CAFs	FAP, MMP2, CTSK
2	239	8	4	121	18	4	0	Myofibroblasts	TAGLN, MYL9, TPM1, TPM2
3	228	65	3	2	12	1	5	CAFs	FAP, MMP2, CTSK
4	214	128	11	2	7	1	5	CAFs	FAP, MMP2, CTSK
5	210	30	14	11	129	1	1	CAFs	FAP, MMP2, CTSK
6	184	14	0	8	28	10	0	Myofibroblasts	TAGLN, MYL9, TPM1, TPM2
7	106	3	6	31	3	2	0	Myofibroblasts	TAGLN, MYL9, TPM1, TPM2
8	79	37	13	0	1	0	27	CAFs	FAP, MMP2, CTSK
9	39	23	12	0	4	0	0	CAFs	FAP, MMP2, CTSK
10	21	1	0	6	2	0	0	Myofibroblasts	TAGLN, MYL9, TPM1, TPM2

Supplementary Table S8. Relative expression of target proteins in validation cohort 3 (The relative intensity is calculated by equal: $R_{sp}=I_{sp} / Mean(I_p)$, R stands for relative expression, I stands for normalized intensity, s stands samples, p stands for protein)

Protein accession	Gene Name	R_{sp}									
		ST31	ST32	ST33	ST34	ST35	DMT31	DMT32	DMT33	DMT34	DMT35
Q12884	FAP	0.08	0.22	0.53	0.08	0.39	1.25	1.88	1.59	1.40	2.58
P01344	IGF2	0.14	0.00*	0.09	0.13	0.64	1.33	1.89	1.40	1.82	2.56
P24844	MYL9	1.92	2.15	0.81	1.14	1.95	0.35	0.39	0.51	0.32	0.46
P49863	GZMK	2.07	2.07	1.75	1.39	1.18	0.60	0.94	0.00*	0.00*	0.00*

* Protein signals are not detected in the MS scan.

Supplementary Table S9. Patient and tumor characteristics at presentation in clinical trial

Patient	CoM-10	CoM-11
Age	51	76
Sex	Female	Female
Tumor location	Eyelid	Eyelid
Tumor thickness	3.4mm	2.2mm
Ulceration	Present	Present
TIL	Brisk	Brisk
Regression	Absent	Absent
Mitotic rate	1	3
Perineural invasion	Present	Present
Vascular invasion	Absent	Absent
Microscopic satellitosis	Absent	Absent
Clinical T category	3	2
N category	0	0
M category	0	0
Sites of distant metastasis	Liver & Brain	Lung
Origin	De Novo	Nevus
Driver mutation	BRAF V600E	BRAF V600E
Samples name	NA	NA
Immune therapy	anti PD-1 (Camrelizumab)	anti PD-1 (Camrelizumab)
Target therapy	anti VEGFR (Apatinib)	anti VEGFR (Apatinib)
Prognosis	Dead	SD

Supplementary Table S10. Patient and tumor characteristics at presentation in clinical cohort

Patient	CoM-01	CoM-02	CoM-03	CoM-04	CoM-05	CoM-06	CoM-07	CoM-08	CoM-09	CoM-10	CoM-11
Age	66	46	56	53	61	51	80	57	55	51	76
Sex	Female	Female	Female	Female	Male	Male	Male	Male	Male	Female	Female
Tumor location	Eyelid	Orbit	Eyelid	Eyelid	Bulbar conjunctiva & Palpebra	Eyelid	Eyelid	Eyelid	Eyelid	Eyelid	Eyelid
Tumor thickness (mm)	8.5	NA	4.5	3.4	3.5	0.8	4	5.1	3.6	3.4	2.2
Ulceration	Present	Absent	Absent	Absent	Present	Absent	Present	Absent	Absent	Present	Present
TIL	Brisk	Brisk	Brisk	Brisk	Brisk	Brisk	Absent	Brisk	Brisk	Brisk	Brisk
Regression	Absent	Absent	Absent	Absent	Absent	Absent	Absent	Absent	Present	Absent	Absent
Mitotic rate	25	2	13	3	2	7	5	11	10	1	3
Perineural invasion	Absent	Present	Absent	Absent	Absent	Absent	Absent	Absent	Absent	Present	Present
Vascular invasion	Absent	Present	Absent	Absent	Absent	Absent	Absent	Absent	Absent	Absent	Absent
Microscopic satellitosis	Absent	Absent	Absent	Absent	Absent	Absent	Absent	Absent	Absent	Absent	Absent
Clinical T category	3	4	3	3	3	3	3	3	3	3	2
N category	0	1	1	0	1	0	0	0	0	0	0
M category	0	0	0	0	1	0	0	0	0	0	0

Sites of distant metastasis	Lung	Lung	Multiple organs	Multiple organs	Liver	Liver & Lumbar	Lung	Multiple organs	Lung	Liver & Brain	Lung
Origin	Nevus	De Novo	De Novo	De Novo	De Novo	Nevus	PAM	PAM	De Novo	De Novo	Nevus
Driver mutation	BRAF V600E	NA	NRAS Q61R	NA	NRAS G12A	BRAF V600E	NA	NA	NA	BRAF V600E	BRAF V600E
Samples name	NA	NA	Mel0909 & Lym0909	NA	Mel0722 & Lym0722	Mel0618	NA	NA	NA	NA	NA
Immune therapy						anti PD-1 (Pembrolizumab)	anti PD-1 (Toripalimab)	anti PD-1 (Toripalimab)	anti PD-1 (Pembrolizumab)	anti PD-1 (Camrelizumab)	anti PD-1 (Camrelizumab)
Target therapy										anti VEGFR (Apatinib)	anti VEGFR (Apatinib)
Prognosis	Dead	Dead	Dead	Dead	Dead	Dead	PD	PD	SD	Dead	SD

Supplementary Table S11. The list of antibodies used in this study

Antibodies	Identifier	Source
VIM	Cat# 10366-1-AP	Protein tech
JUN	Cat# 3270S	Cell Signaling
MLANA	Cat# ab210546	Abcam
EGR1	Cat# ab300449	Abcam
CD31	Cat# 11265-1-AP	Protein tech
VEGFR	Cat# 2479S	Cell Signaling
MYL9	Cat# 15354-1-AP	Protein tech
CD8	Cat# 66868-1-Ig	Protein tech
GZMK	Cat# 98946S	Cell Signaling
SELL	Cat# 58225S	Cell Signaling
DAPI	Cat# 28718-90-3	Sigma-Aldrich
FAP	Cat# BMS168	Invitrogen
